# Reproductive behavior drives female space use in a sedentary Neotropical frog

**DOI:** 10.7717/peerj.8920

**Published:** 2020-04-17

**Authors:** Marie-Therese Fischer, Max Ringler, Eva Ringler, Andrius Pašukonis

**Affiliations:** 1 Department of Evolutionary Biology, University of Vienna, Vienna, Austria; 2 Department of Behavioral and Cognitive Biology, University of Vienna, Vienna, Austria; 3 Messerli Research Institute, University of Veterinary Medicine Vienna, Medical University Vienna, University of Vienna, Vienna, Austria; 4 Department of Biology, Stanford University, Stanford, CA, United States of America

**Keywords:** *Allobates femoralis*, Home range, Poison Frog, Tracking, Fine-scale movement, Reproductive behavior, Space-use

## Abstract

Longer-range movements of anuran amphibians such as mass migrations and habitat invasion have received a lot of attention, but fine-scale spatial behavior remains largely understudied. This gap is especially striking for species that show long-term site fidelity and display their whole behavioral repertoire in a small area. Studying fine-scale movement with conventional capture-mark-recapture techniques is difficult in inconspicuous amphibians: individuals are hard to find, repeated captures might affect their behavior and the number of data points is too low to allow a detailed interpretation of individual space use and time budgeting. In this study, we overcame these limitations by equipping females of the Brilliant-Thighed Poison Frog (*Allobates femoralis*) with a tag allowing frequent monitoring of their location and behavior. Neotropical poison frogs are well known for their complex behavior and diverse reproductive and parental care strategies. Although the ecology and behavior of the polygamous leaf-litter frog *Allobates femoralis* is well studied, little is known about the fine-scale space use of the non-territorial females who do not engage in acoustic and visual displays. We tracked 17 females for 6 to 17 days using a harmonic direction finder to provide the first precise analysis of female space use in this species. Females moved on average 1 m per hour and the fastest movement, over 20 m per hour, was related to a subsequent mating event. Traveled distances and activity patterns on days of courtship and mating differed considerably from days without reproduction. Frogs moved more on days with lower temperature and more precipitation, but mating seemed to be the main trigger for female movement. We observed 21 courtships of 12 tagged females. For seven females, we observed two consecutive mating events. Estimated home ranges after 14 days varied considerably between individuals and courtship and mating associated space use made up for ∼30% of the home range. *Allobates femoralis* females spent large parts of their time in one to three small centers of use. Females did not adjust their time or space use to the density of males in their surroundings and did not show wide-ranging exploratory behavior. Our study demonstrates how tracking combined with detailed behavioral observations can reveal the patterns and drivers of fine-scale spatial behavior in sedentary species.

## Introduction

The decision to stay or to move to other places is crucial for many aspects of an animal’s life including foraging, territorial behavior, mating strategies and responses to predators (Brown & Orians, 1970; Swingland & Greenwood, 1983; Murray & Fuller, 2000; Wells, 2007; Newton, 2008; Sinsch, 2010; Brown, Morales & Summers, 2010). To uncover which aspects drive an animal’s movement, it is necessary to monitor its behavior across all relevant spatio-temporal scales, as causes and patterns of distinct movement phases that build up a lifetime track might differ significantly (Brown & Orians, 1970; Fryxell et al., 2008). For example, many passerine birds of the Northern Hemisphere will forage in their close surrounding, learn about resources by exploring their neighborhood, migrate hundreds of kilometers south to outlast winter, and return to their natal area to breed (Newton, 2008). All movement is costly and bears substantial risks such as increased energy expenditure, elevated conspicuousness to predators and exposure to harsh climatic conditions, all of which can favor a sedentary lifestyle (Bell, 2012; Dingle, 2014). Amphibians are often considered to be the most sedentary of terrestrial vertebrates and long-term fidelity to patches of suitable habitat is common in many species (Wells, 2007).

Most information on spatial behavior in anurans comes from capture-mark recapture (CMR) studies (Wells, 2007; Walston & Mullin, 2008; Brown, Morales & Summers, 2009; Bull, 2009; Weinbach et al., 2018). With this method, any movement between recaptures rests unexplored, confining estimation of spatial and temporal characteristics to larger scales. Understanding fine-scale movement of amphibians requires more continuous tracking, in particular for sedentary species. To date, the vast majority of studies on amphibian space use focused on migratory behavior of temperate amphibians characteristic of the seasonal and lifetime spatio-temporal scale, e.g., the synchronized mass migration from winter habitats to breeding sites, return migrations of adults between different parts of the habitat or dispersal of juveniles (e.g., Heusser, 1968; Sztatecsny & Schabetsberger, 2005; Sinsch et al., 2012) (for reviews see Richards, Sinsch & Alford, 1994; Sinsch, 2010; Pittman, Osbourn & Semlitsch, 2014; Sinsch 2014). Tropical amphibians show much more diverse reproductive and spatial behaviors, such as long-term site fidelity, territoriality, courtship, and offspring transport (Wells, 2007; Summers & Tumulty, 2014) but very few studies have quantified the fine-scale movements of tropical amphibians (Brown et al., 2006; Oliveira et al., 2016; Ward-Fear, Greenlees & Shine, 2016; Pettit, Greenlees & Shine, 2017).

Poison frogs (Dendrobatidae; sensu (AmphibiaWeb, 2020); but see also (Grant et al., 2017; Guillory et al., 2019) are a well-studied group of small Neotropical frogs with parental care (Grant et al., 2006; Lötters et al., 2007; Wells, 2007). The challenges of when, where and how to care for offspring require complex spatio-temporal strategies, making poison frogs ideal organisms to study ecological aspects of movement and orientation (Sinsch, 1990; Sinsch, 2010; Brown, Morales & Summers, 2010; Pittman, Osbourn & Semlitsch, 2014). Although parenting strategies in this group of frogs have attracted a considerable amount of research (e.g., Weygoldt, 1987; Summers et al., 1999; Summers & McKeon, 2004; Poelman & Dicke, 2007; Wells, 2007; Brown, Morales & Summers, 2008; Brown, Morales & Summers, 2010; Ringler et al., 2013; Summers & Tumulty, 2014; Roland & O’Connell, 2015 Dugas et al., 2016; Schulte & Summers, 2017), associated fine-scale movement patterns and factors affecting them have rarely been quantified and remain poorly understood. Poison frogs are diurnal and mostly show long-term site fidelity or territoriality (Pröhl, 2005; Wells, 2007), displaying their whole behavioral repertoire in relatively small areas. Space use in this clade is shaped by various factors, such as parental care strategies (Brown, Morales & Summers, 2009), parental state (Haase & Pröhl, 2002), number of surrounding mating partners (Folt, Donnelly & Guyer, 2018), distribution of reproductive sites (Donnelly, 1989; Pröhl & Berke, 2001) or abundance of food (Pough & Taigen, 1990). While male site fidelity and long-distance movements in species with uniparental male care is closely linked to caretaking duties (Brown, Morales & Summers, 2009; Ursprung et al., 2011; Beck et al., 2017), movement patterns and reasons for site-fidelity are not well understood for the polygamous females. In this study, we use tracking to investigate fine-scale movement of female Brilliant-Thighed Poison Frogs (*Allobates femoralis* (BOULENGER 1883)).

*Allobates femoralis* is a well-studied species particularly suited to investigate fine-scale spatial behavior of females: they are large enough to fit tags for tracking (Pašukonis et al., 2014; Beck et al., 2017) and frogs can be identified by their unique ventral pattern, facilitating monitoring of individuals via CMR (Ringler, Mangione & Ringler, 2014). Furthermore, long- and short-term population studies with a CMR approach have revealed many aspects of *A. femoralis* behavior and ecology (Ringler, Ursprung & Hödl, 2009; Ursprung et al., 2011; Ringler et al., 2013; Ringler et al., 2014).

During the reproductive season, males establish and defend territories which are advertised by calling from elevated structures such as palm leaves, logs or branches (Weygoldt, 1980; Narins, Hödl & Grabul, 2003; Hödl, Amézquita & Narins, 2004; Amézquita et al., 2006). Females are attracted by calling males and deposit clutches in the male’s territory after a prolonged courtship (Weygoldt, 1980; Roithmair, 1994; Montanarin, Kaefer & Lima, 2011; Stückler et al., 2019). Males typically transport tadpoles from the leaf litter clutch to water bodies outside of the territory (Lescure, 1976; Ringler et al., 2013; Ringler et al., 2018), but females take over the duty when fathers disappear (Ringler et al., 2015). Tracking has been successfully applied to study spatial behavior and navigation in this species. For example, telemetry was used to quantify movements associated with tadpole transport (Beck et al., 2017; Pašukonis et al., 2017) or to demonstrate that *A. femoralis* males return to their home territory from several hundred meters after translocation (Pašukonis et al., 2014).

Female *A. femoralis* do not defend territories but show site fidelity and seem to retreat to small perches from where they commute to neighboring males for courtship and mating (Ringler, Ursprung & Hödl, 2009; Ringler et al., 2012). They do not engage in acoustic and visual displays and are therefore harder to localize and more challenging to survey than calling territorial males. Consequently, little is known about their spatial and temporal movement patterns, time budget or factors influencing their movement. The aim of this study was to fill the knowledge gap in fine-scale spatial behavior of female *A. femoralis* by using individual tracking to address the following questions:

1. What is the daily activity pattern of female Brilliant-Thighed Poison Frogs?
2. Which factors influence female movement?
3. What are the metrics of female space use and how do females budget their time?

We integrate tracking data with seasonal CMR-based monitoring in *A. femoralis* and provide the first fine-scale spatial analysis of female site fidelity in polygamous poison frogs.

## Materials & Methods

### Ethics statement

All animal handling procedures were conducted in strict accordance with current French and EU law and followed the guidelines of the Association for the Study of Animal Behavior (ASAB, 2018) for the treatment of animals in behavioral research and teaching. Our study was approved by the Animal Ethics and Experimentation Board of the Faculty of Life Sciences, University of Vienna (approval number: 2016-003, PI: Andrius Pašukonis). The permission to conduct fieldwork was provided by the prefect of the Guiana region (approval document ‘Arrete no 011-44 /DEAL/SMNBSP/BSP du 19/07/2011’). In addition to this permit, all protocols for fieldwork were approved by the scientific committee of the station Saut Pararé (managed by the Centre Nationale de la Recherche scientific, CNRS). The decision of the scientific committee was communicated as oral agreement by the technical director of the station, Philippe Gaucher (representing CNRS) to Eva and Max Ringler (representing the University of Vienna).

### Study site & study population

The study was conducted on a 4.6 ha river island located in the immediate vicinity of the CNRS research station Saut Pararé (4°02′N/52°41′W), within the Nature Reserve Les Nouragues in a tropical rain forest in French Guiana (Bongers et al., 2011). The study area consists of primary terra-firme forest with seasonally flooded areas on the edges of the island. This island in the Arataye River was originally not populated by our study species. The experimental population of *A. femoralis* was established in 2012, when 1,800 tadpoles from the adjacent mainland population were introduced into artificial pools on the river island (Ringler, Mangione & Ringler, 2014). During the time of our study, the frogs primarily relied on an array of 13 artificial pools (volume ∼12 l, inter-pool distance ∼20 m). A detailed map of the island, including living and dead trees (DBH ≥10 cm), palms, fallen logs, larger branches, trails, waterbodies and artificial pools is available and constantly updated (Ringler et al., 2014).

### Data collection

#### Monitoring

Individuals were monitored during their reproductive season between 24 January and 14 April 2016 using a CMR approach. For this purpose, we systematically surveyed the entire island to capture, map and identify as many frogs as possible. The procedure was repeated throughout the monitoring period with the attempt to sample the whole population and to gain information on the spatial distribution and stability of individual territories over time. The frogs were captured with transparent plastic bags, sexed by the presence (male) or absence (female) of the vocal sac and their unique ventral pattern was photographed for subsequent individual identification using the pattern matching software Wild-ID (Bolger et al., 2012). Spatial location, sex, current behavior and picture number were noted on a tablet PC (WinTab 9, Odys, Willich, Germany) using a high-resolution map of the island in the mobile GIS software ArcPad 10.2 (ESRI, Redlands, CA, USA).

#### Tracking frogs using a harmonic direction finder

**Figure 1:**
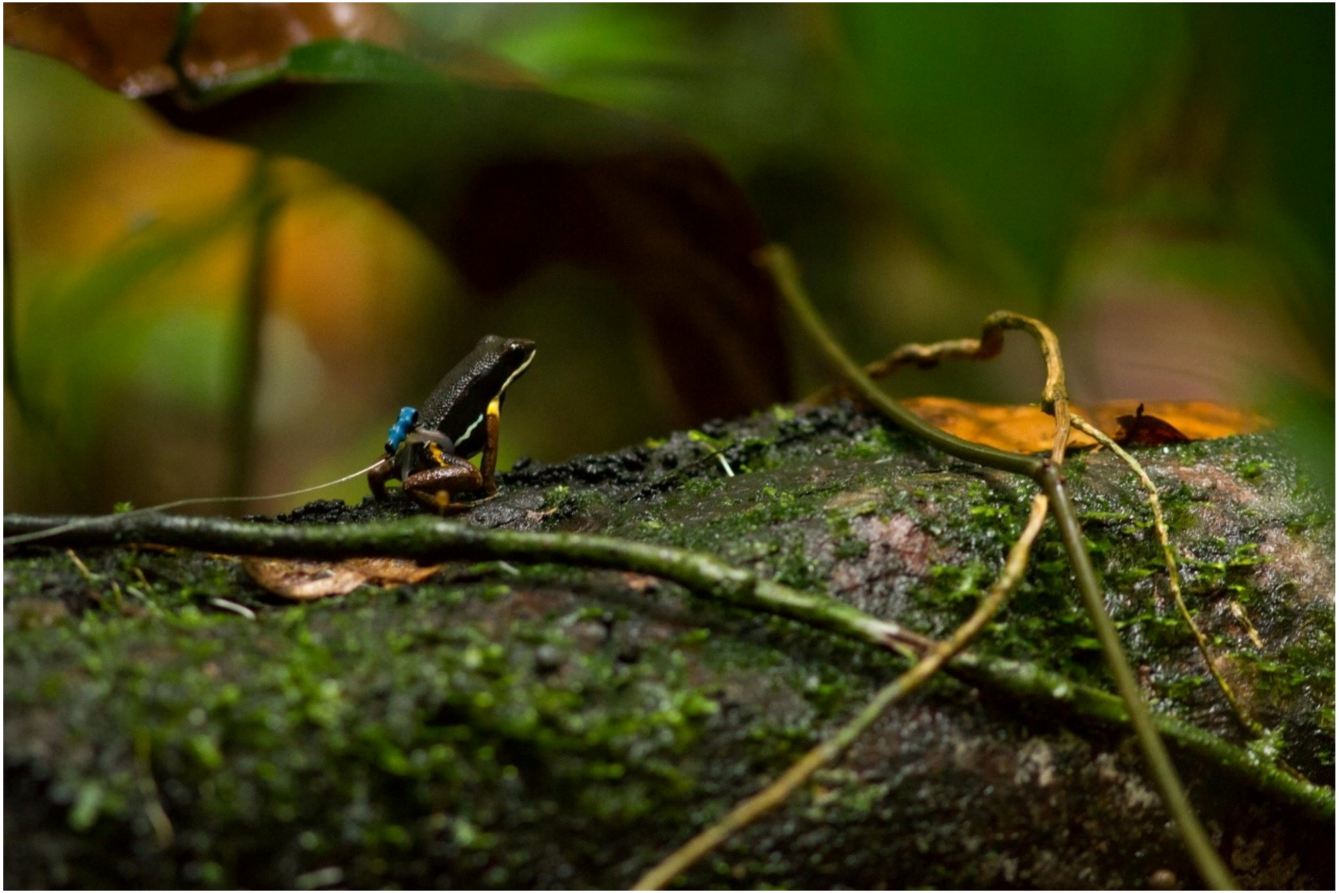
Tag attachment. Female *A. femoralis* equipped with a miniature reflector tag for HDF telemetry, fixed to a waistband that is fitted to the size of the individual. Photo credit: Andrius Pašukonis.

We tracked individual frogs between 8 February and 20 March 2016 by equipping them with miniature tags that reflect the signal emitted from a harmonic direction finder (RECCO R8, Recco AB, Lindigö, Sweden). We either tagged females immediately after an observed clutch deposition or after opportunistic encounters. We identified frogs by their individual ventral pattern and fitted miniature tags using a silicon-tube waistband with an additional strap between the hind legs to prevent the tag from rotating (Fig. 1). The tags consisted of a reflector diode which was color-coded, sealed with air-dry rubber (2 mm diameter) and connected to a T-shaped dipole antenna made of thin multi-strand coated beading wire. The long end (∼12 cm) dragged freely behind the frog while the short end (∼2 cm) was secured inside the waistband (as descibed in Pašukonis et al., 2013; Beck et al., 2017). We tracked up to six individuals simultaneously between 6 and 17 days. We relocated the females every 60 min between 07.30 h and 18.30 h during their diurnal activity period and recorded their position. We chose a lose fit for the waistbands to avoid egg binding. We checked for eventual tag-induced injuries every third morning, removed the tag if any wounds were visible and also used later CMR recaptures to check for wounds. Because of the lose waistband fit, some females lost their tags. In most cases, we were able to find and retag the same individual. The mean number of recaptures per individual during the tracking was four (*n* = 17). Of the 17 tagged females, one was found dead after six days without any signs of predation, one was found dead in proximity to a large spider, two lost the tag and could not be recaptured to continue tracking, one was untagged because of tag-induced injury of the skin, and one female disappeared with the tag. All other 11 females were untagged after tracking.

For each data point, we additionally noted any observed behavior (e.g., social interactions, courtship, tadpole transport, egg deposition, feeding). Courtship behavior was assigned when the female was following a male producing a courtship call (Stückler et al., 2019). After each courtship, we recorded the exact location of the oviposition site and captured and identified the mating partner.

### Data analysis

#### Software & statistics

We processed spatial data (WGS84/UTM zone 22N) in ArcMap 10.4 (visualization, distance measurements, creation of Minimum Convex Polygons), QGIS 3.2.3 (creation of Voronoi polygons, extraction of X and Y coordinates for each data point) (QGIS Development Team, 2019) and R 3.5.3 using RStudio 1.2.1335 (RStudio Team, 2015) with the packages ‘adehabitatLT’ (function ‘as.ltraj’, distance between consecutive relocations, trajectory analysis) and ‘adehabitatHR’ (functions ‘mcp’ and ‘KUD’, home range analysis) (Calenge, 2006). All statistical comparisons and illustrations were done in R using the packages ‘stats’ (functions ‘hist’ for histogram, ‘t.test’ for a paired *t*-test, ‘shapiro.test’ Shapiro test, ‘cor.test’ for Spearman correlation, ‘lm’ for linear regression) (R Core Team, 2019), ‘lawstats’ (function ‘levene.test’ for Levene test) (Gastwirth et al., 2019), ‘lme4’ (function ‘lmer’ for a linear model) (Bates et al., 2015). The distribution of all sets of data was checked visually with a histogram and statistically using a Shapiro test. All illustrations were edited and compiled in Inkscape 0.92.3 (Harrington et al., 2004–2005).

#### Female activity patterns

We determined the reproductive status of each female daily. Courtship in *A. femoralis* typically spans two days: it starts on the first day with a prolongated ‘courtship march’ where the female follows a male emitting courtship calls (Stückler et al., 2019; this study). The couple typically spends the nights under a leaf. On the morning of the second day, they continue the courtship march for a short while before they choose a site for mating and oviposition. We recorded days without any reproductive behavior as ‘no courtship’ (NC).

For each female we calculated a mean distance per hour for NC days (n_females_ = 17), the day of courtship initiation (n_females_ = 12) and the mating day (n_females_ = 12). Mean values from all females were used to calculate an average distance per hour of the day on NC days, days of courtship initiation and mating days.

#### Female movement in relation to weather conditions

We calculated daily movement distances from consecutive relocations of each female. We obtained rainfall data in 30-minute-intervals from the ‘Nouraflux’ station located on the ‘Canopy Operating Permanent Access System’ (COPAS) (Gottsberger, 2017) near our study area. Precipitation was summed over the whole day (including nighttime) as rainfall during the night contributes to the relative humidity during the daytime and might influence the movement of the frogs. We recorded temperature every 15 min with a logger (HOBO Pro v2 U23) placed approximately 30 cm above ground level in the center of the island. Temperature was averaged over the daylight time (between 06.30 h and 18.30 h), which approximately corresponds to the active period of the frogs. All tracked females (*n* = 17) were included in the analysis. We tested the correlation between daily movement (m) and average diurnal temperature (^∘^ C) as well as average daily rainfall (mm) using a Spearman correlation.

#### Female movement in relation to reproductive state

In this analysis we included only females for which courtship was observed (*n* = 12) and calculated differences between days with and without reproductive behavior with a linear mixed model using the function ‘lmer’ (R package ‘lme4’). This approach allows to include the identity of the females as a random factor. For every female, we calculated the mean distance moved on days with courtship and mating and NC days. The reproductive state was included into the mixed model as fixed factor, while the identity of the female (‘ID’) was included as a random factor. In addition, we compared the mean distance moved on the day of courtship initiation to the mean distance moved on mating days with a paired *t*-test (function ‘t.test’, R package ‘stats’). The variance between the respective grouping variables (days with courtship or mating and NC or courtship and mating) was analyzed with a Levene test.

#### Algorithms estimating home range (HR)

We estimated the HR with two methods to improve the robustness of our analysis: minimum convex polygons with 5% of outliers removed (MCP95) for comparisons with previous studies of poison frog space use, and kernel utilization distributions with 5% of outliers removed (KUD95) for a more detailed assessment of space use.

HR calculations were performed in R using the functions ‘mcp’ and ‘KUD’ (package ‘adehabitatHR’, parameters: grid = 200, extend = 1, h = href, same for all = FALSE). Differences in HR area between the two estimators were assessed with a paired *t*-test.

Linear regressions between tracking duration and HR size (MCP95 and KUD95; *n* = 17) were calculated in R using the function ‘lm’ (R package ‘stats’). We repeated the regression analysis with a reduced dataset including only females that were tracked for at least 14 days (*n* = 9).

To explore how HR changes over time, we calculated daily cumulative HRs with both methods. The minimum number of nine points required to calculate KUDs with ‘adehabitat HR’ was reached after the second day of tracking for all females. Due to the rather low number of points per female in the beginning, HR was typically vastly overestimated with KUD95 on day two compared to calculations including three or more tracking days. We therefore report the cumulative KUD HR starting from tracking day three.

#### HR in relation to reproductive status

To identify changes in the HR associated with reproduction we calculated the mean change in cumulative HR area for courtship, mating, and NC days. Females were included into the analysis when the complete reproductive sequence (courtship and oviposition) was observed (*n* = 11). The reproductive status was included into the linear mixed model as fixed factor, while the identity of the female (‘ID’) was included as a random factor. The variance between the grouping variables was analyzed with a Levene test.

In a second analysis we estimated the HR of each female with MCP95 and KUD95 using the full as well as a reduced dataset, where we excluded days with courtship and mating. We compared HR area with and without days with reproductive activity for each female and determined the mean percentage of area difference between the two datasets.

#### Female centers of use

Based on Wilson et al. (2010), we defined ‘centers of use’ as areas with a higher density of use than other areas within a frog’s HR. We visually inspected consecutive 5% isopleths (5%–100%) of the KUD to distinguish areas with high use for each female. The 30% isopleth was found to distinguish the highest number of centers per frog while correlating well with clusters of relocation points, indicating areas of higher use. Therefore, we defined the KUD30 area as ‘centers of use’ for the females in our study.

#### Female trajectories

For each female we calculated the total tracking time as well as the actual daytime tracking time excluding the night hours where frogs typically rest under a leave without moving. We calculated the daily movement distance as the sum of all consecutive distances between the relocations of each frog and computed the mean, minimum, and maximum daily distances as well as the total distance for the full tracking. From the individual measures we calculated the average total/daily distance across all females (*n* = 17). To determine the average distance moved per hour we divided the total distance by the total hours of tracking for each female and averaged over all individuals (*n* = 17). The distance between the two most distant points of the trajectory was extracted in ArcMap 10.4.

For the illustration of the female trajectories, we overlaid their paths with estimations of surrounding male territories, based on Voronoi tessellations of male CMR points. This method partitions an area (the island) into polygons, based on equidistant midlines between pairs of points (Voronoi, 1908). The Voronoi polygons were calculated using the function ‘Voronoi tessellation’ in QGIS using the CMR points of all males. To account for territory dynamics, for each respective female, only the five most recent capture points of each male prior to the end of the tracking session were included into the analysis. As the shore of the island is rather steep and not occupied by *A. femoralis*, we truncated the outer territories midway between a male’s outermost locations and the island shore by adding the vertices of the island outline to the points used for the Voronoi tessellation.

#### Time budget

We further analyzed the female trajectories in relation to the centers of use by assigning each tracking point of a female to one in the following movement categories: relocations in the centers of use (C1–C3), movements of unknown purpose outside the centers of use not directly followed by a courtship event (sallies), male directed movement followed by a courtship (pre-mating movement) and movement from the egg deposition site until reaching a center of use (post-mating movement). The initial movement after tagging, before reaching a center of use, was treated as a distinct category as we could not assign this movement to sally or post-mating movement or to another center of use that was not identified during the tracking. Initial movement was not assigned if the first capture point of the female was already located inside a center of use. We calculated the percentage of each category by relating the number of points per behavior to the total number of tracking points collected for the respective female.

### Influence of male density on female spatial behavior

To estimate male density we calculated a KUD based on the individual male centroid points calculated in ArcGIS from all male CMR points that were not associated with tadpole transport. We used the ‘Kernel Density’ function in ArcGIS with a raster cell size of 0.2 ×  0.2 m and a search radius of 35 m (cf. Ringler, Hödl & Ringler, 2015). Average male density was extracted for each female using the ‘Zonal Statistics as Table’ tool in ArcGIS) with the female KUD30 shapefile for selection.

We calculated linear regressions to correlate the mean male density with (1) the size of the female centers of use, (2) the number of female perches and (3) the percent of time spent on sallies by the respective female.

#### Revisits of previous egg deposition sites

We assessed whether females revisit their previous clutches by calculating the distance of each tracking point to the first observed egg deposition for all females with the ‘Distance Matrix’-tool in QGIS. An approach of <2.5 m to the first clutch was defined as a revisit. We analyzed if females approached their old clutches, on which occasion, and after which time. Additionally, we calculated the distance between consecutive clutches from one female using the ‘Measure Line’ tool in QGIS.

#### Comparison of tracking and CMR home ranges

We compared HR calculations from tracking (up to 17 consecutive days) and the CMR dataset (up to 71 days) to assess the stability of the HR over periods exceeding the tracking time. We used the MCP method with 100% of data points (MCP100) instead of MCP95 because of the lower number of capture points in the CMR dataset. Only tracked females with at least four CMR points were included in this analysis. We determined the overlap between the MCPs from tracking and CMR data per female, using the ‘intersect’ tool in QGIS. As the CMR MCPs were usually smaller than the MCPs from tracking, we reported the intersected area as percentage of the tracking area. We also compared the averaged HR size (MCP100) of all females represented by 4 or more capture points (*n* = 23) to the averaged HR size of all tracked females (*n* = 17).

#### Behavioral observations

We observed and recorded general behavior and examined whether tagged females show the same behavioral repertoire than females without a tag. We reviewed reports on natural behavior of *A. femoralis* females from the literature (Table S1) and compared them to the presence/absence of these behaviors in females equipped with a tag. As reproductive traits differ considerably between different *A. femoralis* populations (e.g., different rate of male rejection during courtship (Stückler et al., 2019)), we focused on behaviors described for the same (e.g., Stückler et al., 2019) or nearby populations.

## Results

During the study period, the *A. femoralis* study population encompassed 85 males and 82 females, as determined with a concurrent CMR monitoring at the field site. We equipped 17 females with a reflector tag and tracked them for 6–17 days. Eleven out of 17 females were captured in 2016 for the first time, five were already identified in 2015 and one in 2014.

### Female activity patterns

**Figure 2:**
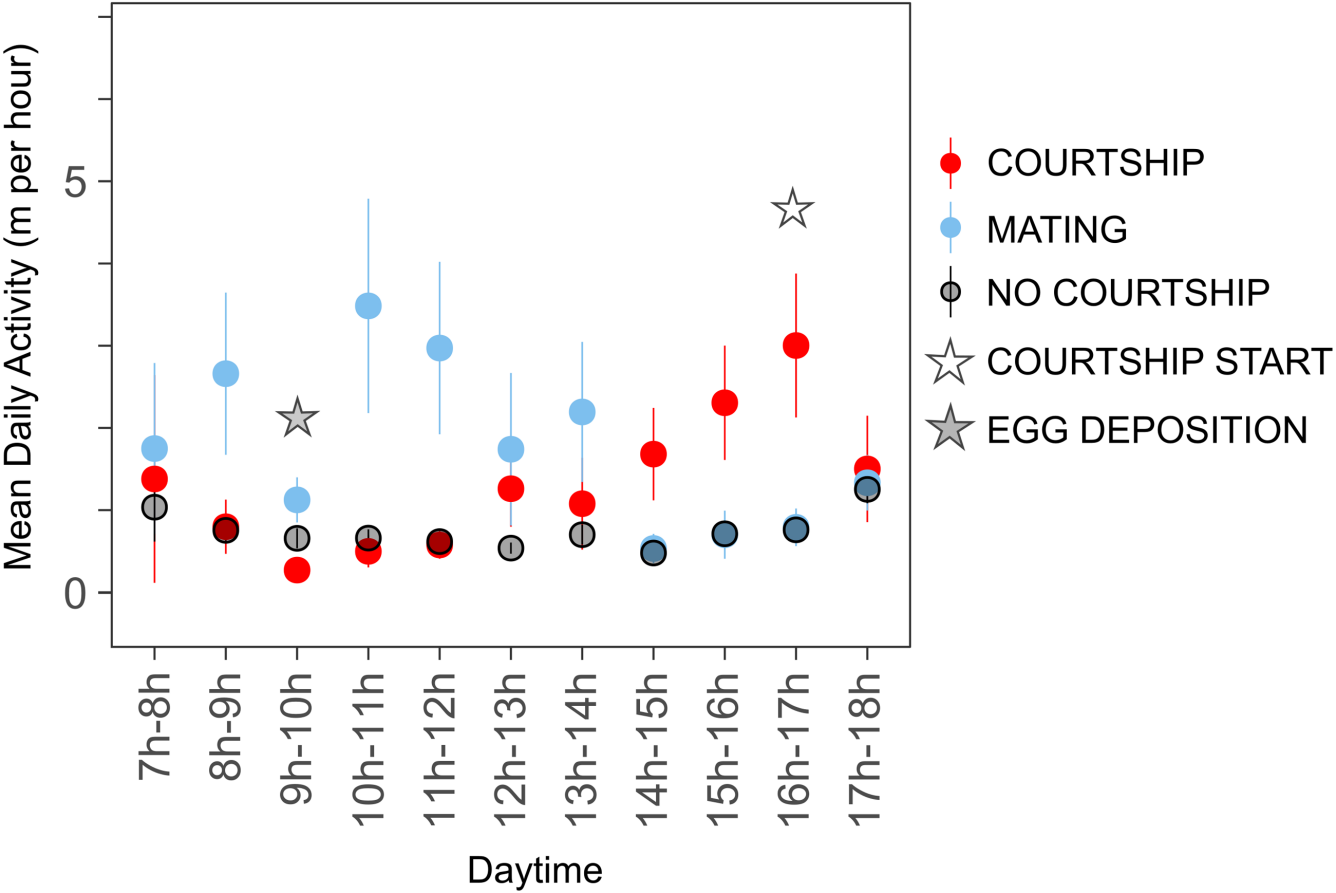
Female activity patterns throughout the day. We calculated the mean distance traveled per hour of the day for each female and averaged it over all study subjects (‘mean daily activity’, n = 17). The mean activity throughout the day is shown for days without courtship (black), on the day of courtship initiation (red) and on the mating day (blue). The most frequent hour of courtship initiation is marked with a hollow star, the most frequent hour of egg deposition is indicated by a grey star.

Females moved on average shorter distances on days without courtship or mating (*n* = 12, mean = 0.75 ± 0.22 m/h, range = 0.48–1.26 m/h) with maximal movement observed between 17.00 h and 18.00 h. On days of courtship initiation, movement speed peaked in the late afternoon between 16.00 h and 17.00 h (*n* = 12, mean = 1.41 ± 0.91 m/h, range = 0.28–3 m/h), while maximal distances were covered between 10.00 h and 11.00 h on mating days (*n* = 12, mean = 1.86 ±  0.96 m/h, range = 0.54–3.49 m/h). Courtship events always started in the afternoon, mostly between 16.00 h and 17.00 h (n_total_ = 15; n_15−16_ = 4, n_16−17_ = 6, n_17−18_ = 5), while eggs were deposited in the morning, mostly between 09.00 h and 10.00 h (n_total_ = 15; n_<9*h*_ = 4, n_9−10_ = 8, n_>10_ = 3). Egg deposition on the mating day is reflected by a sudden decrease in movement (Fig. 2).

### Female movement in relation to weather conditions

Daily distances moved by female *A. femoralis* showed a significant correlation with both, temperature during day (^∘^C) and cumulative rainfall per day (mm). While females moved less on days with higher mean temperatures (Spearman’s rank correlation; rho = −0.35, slope = −0.58, *p* = 0.001), daily movement increased slightly on days with higher mean precipitation (Spearman’s rank correlation; rho = 0.36, slope = 0.02, *p* = 0.002) (Fig. S1).

### Female movement in relation to reproductive state

**Figure 3:**
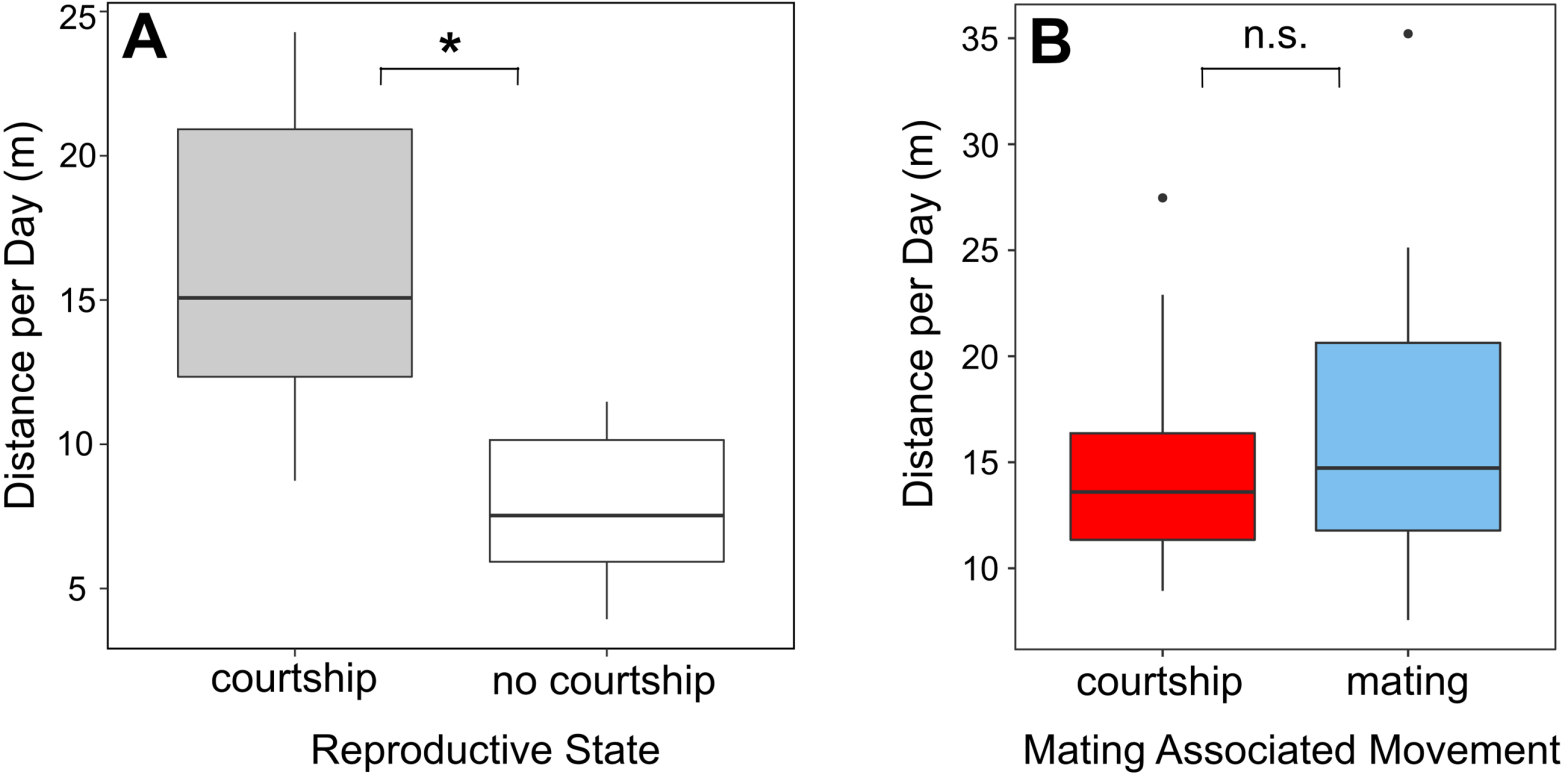
Movement in relation to reproductive behavior. (A) Females moved significantly more on days associated with reproductive events compared to days without courtship or mating (Linear mixed model, *p* = 0.0002). (B) Mating associated movement didn’t differ between the day of courtship start (red) and the mating day (blue) (paired *t*-test, *p* = 0.41).

Females of *A. femoralis* moved significantly longer distances on days with than on days without courtship or mating (Linear mixed model, *n* = 12, estimate = −8.53, SE = 1.59, *df* = 11, t = −5.37, *p* = 0.0002) (Table 1, Fig. 3A). Distances traveled on the day of courtship and on the day of mating did not differ significantly (paired *t*-test, *n* = 11, *t*= −0.85, *df* = 10, *p* = 0.41) (Fig. 3B).

**Table 1:** Trajectory and time budget of *A. femoralis* females. We quantified distances traveled by female frogs, defined centers of more intense use and analyzed the female trajectory with respect to the time spent in centers of use, on sallies to the surrounding and on courtship and mating. Distances indicated for ‘total distance traveled’ were not corrected for tracking duration.

**Space use parameter**	**Descriptive statistics**	
*Total distance traveled (m)*	Average ± SD	111.3 ± 64.3
	Range	18.3–227
*Distance-traveled-per-day (m)*	Average ± SD	8 ± 3.5
	Range	2.6–14.4
*Distance-traveled-per-hour (m)*	Average ± SD	1 ± 0.4
	min_mean_- max_mean_	0.3–1.76
	max	24.03
*Distance traveled on courtship and mating days (m)*	Average ± SD	16.3 ± 5.6
	Range	8.7–24.3
*Distance traveled on days without courtship or mating (m)*	Average ± SD	7.8 ± 2.5
	Range	3.9–11.5
*Most distant points of individual trajectory (m)*	Average ± SD	19.7 ± 9.5
	Range	6.1–40.0
*Centers of use*	Range	1–3
	n_1center_	12
	n_2centers_	3
	n_3centers_	2
	Average ± SD% of time spent (Range)	61.9 ± 10.1 (46–79.8)
*Sallies*	Average ± SD	4.3 ± 2.9
	Range	0–12
	Average ± SD% of time spent (Range)	9.1 ± 7.9 (0–24.6)
*Courtship and mating*	Average ± SD% of time spent (Range)	9.1 ± 7.9 (0–24.6)

### Female home range

The HR areas covered by female *A. femoralis* after the complete tracking period (*n* = 17) and after an equivalent number of tracking days (14 days, *n* = 9) are given in Table 2.

**Figure 4:**
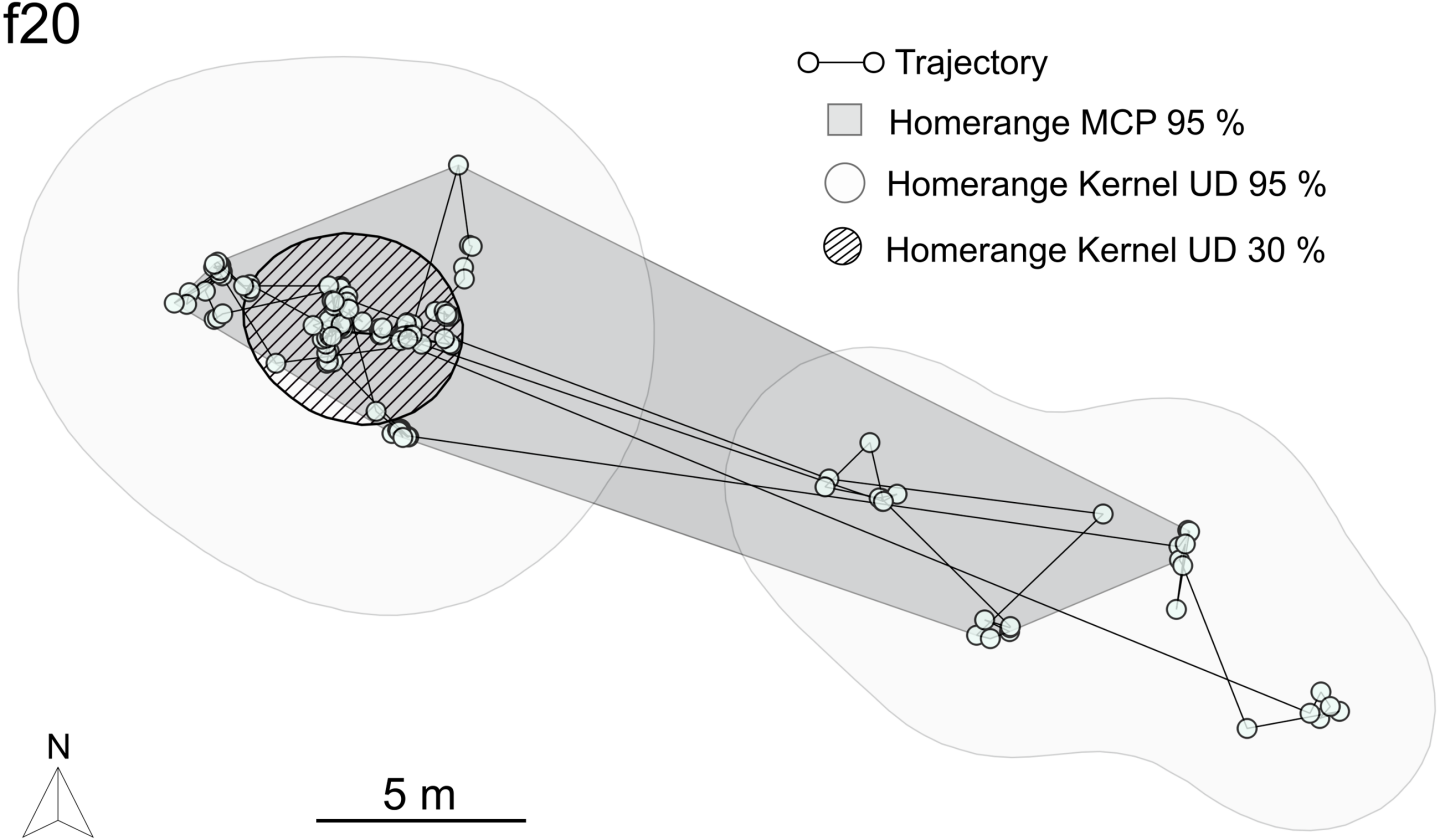
Home range estimation. Datapoints of a representative female (f20, 17 tracking days) are mapped to visualize the two applied models of HR estimation: the more conservative Minimum Convex Polygon calculation (MCP95) is shown dark grey while the HR estimation calculated with KUD95 is depicted in light grey. The KUD30 area was defined as center of use (striped).

Both HR estimates (MCP95 and KUD95, Fig. 4) showed a significant correlation between the covered area and the total tracking time. However, no significant differences between area and tracking time were found if only females tracked for 14 days or longer were included in the analysis (Table S2). The size of female centers of use (KUD30) was weakly correlated with total tracking time. The correlation was not significant if only females tracked for 14 days or longer were included into the analysis (Table S2).

A larger HR was consistently assigned by the KUD95 estimation than by MCP95 (Table S3). At the 95%-isopleth, the HR area of the MCP was on average 56.7% smaller than the KUD estimate.

For both estimators, HR was on average ∼30% smaller when excluding days with courtship and mating (*n* = 11) (Table 2).

### Home range in relation to reproductive behavior

Independent of the HR estimation, the change in female cumulative HR was significantly higher on days when courtship or mating was observed as compared to days without courtship or mating (linear mixed model, *n* = 11; MCP95: estimate = −10.4, SE = 4.421, t = −2.363, *p* = 0.0397; KUD95: estimate = −24.7, SE = 11.53, *t* =  − 2.14, *p* = 0.044). Accordingly, alterations in the estimated HR of the respective females were mostly (Fig. S2A, S2C, S2D–S2F)–but not exclusively (Figs. S2A, S2B, S2E)—associated with reproduction (courtship or mating). Most females showed the highest change in HR directly on days with reproduction (Figs. S2A, S2C, S2D). One female was observed initiating a second mating immediately after failed egg deposition with her first partner (f14, Fig. S2D).

**Table 2:** Home range metrics of female *A. femoralis*. MCP95 and KUD95 estimates of HR metrics of A. femoralis females from 3 datasets are shown. We calculated ‘cumulative HR’ and ‘center of use’ from the complete dataset without correcting for different tracking durations (1). We calculated the ‘HR after 14 days’ to compare the area covered by different females after an equivalent number of tracking days (2). We determined the ‘HR excluding courtship and mating’ from a reduced dataset to quantify the effect of reproduction on the female HR (3).

**Home range metric**	**Estimator**	**N**	**Average** ± SD	**Range (tracking time)**
Cumulative HR (m^2^*)*	MCP95	17	107.4 ± 108.7	6.3 (38 h)–419.1 (148.1 h)
Cumulative HR (m^2^*)*	KUD95	17	215.3 ± 180.9	30.3 (60 h)–657.6 (148.1 h)
Centers of use (m^2^*)*	KUD30	17	20.7 ± 18.8	2.4–66
HR after 14 days (m^2^*)*	MCP95	9	123.07 ± 88.85	24.8–285.2
HR after 14 days (m^2^*)*	KUD95	9	236.2 ± 172.5	59.7–564.9
HR excluding courtship and mating days (m^2^*)*	MCP95	11	92.5 ± 97	18.3–277.9
			(69.4 ± 32% of cumulative HR)	
HR excluding courtship and mating days (m^2^*)*	KUD95	11	179.5 ± 134.9	32–412.5
			(72 ± 26.7% of cumulative HR)	

### Female trajectories and time budget

We tracked females for an average of 108.7 h during their daylight active period between 07.30 h and 18.30 h (*n* = 17; SD = ± 39.2 h; range = 38.0–157.7 h); the average tracking duration including night time was 11.6 days (mean = 279.4 ± 102.3 h). Nights were spent immobile under a leaf and were excluded from further calculations. Distances traveled by females during tracking are listed in Table 1. The fastest recorded movement of 24 m per hour was related to a subsequent mating.

Females had up to three centers of use but most of them returned to one center during the tracking. Centers were on average 9.4 m apart (*n* = 9, SD = ±3.6 m, range = 3.4–14.2 m). The number of sallies to the surrounding varied between 0 and 12 (Table 1, Fig. 5, Figs. S3–S7). All females deposited their eggs outside of their center of use (Fig. 5, Figs. S3– S5, S7, S9–S12, S15–S17).

**Figure 5:**
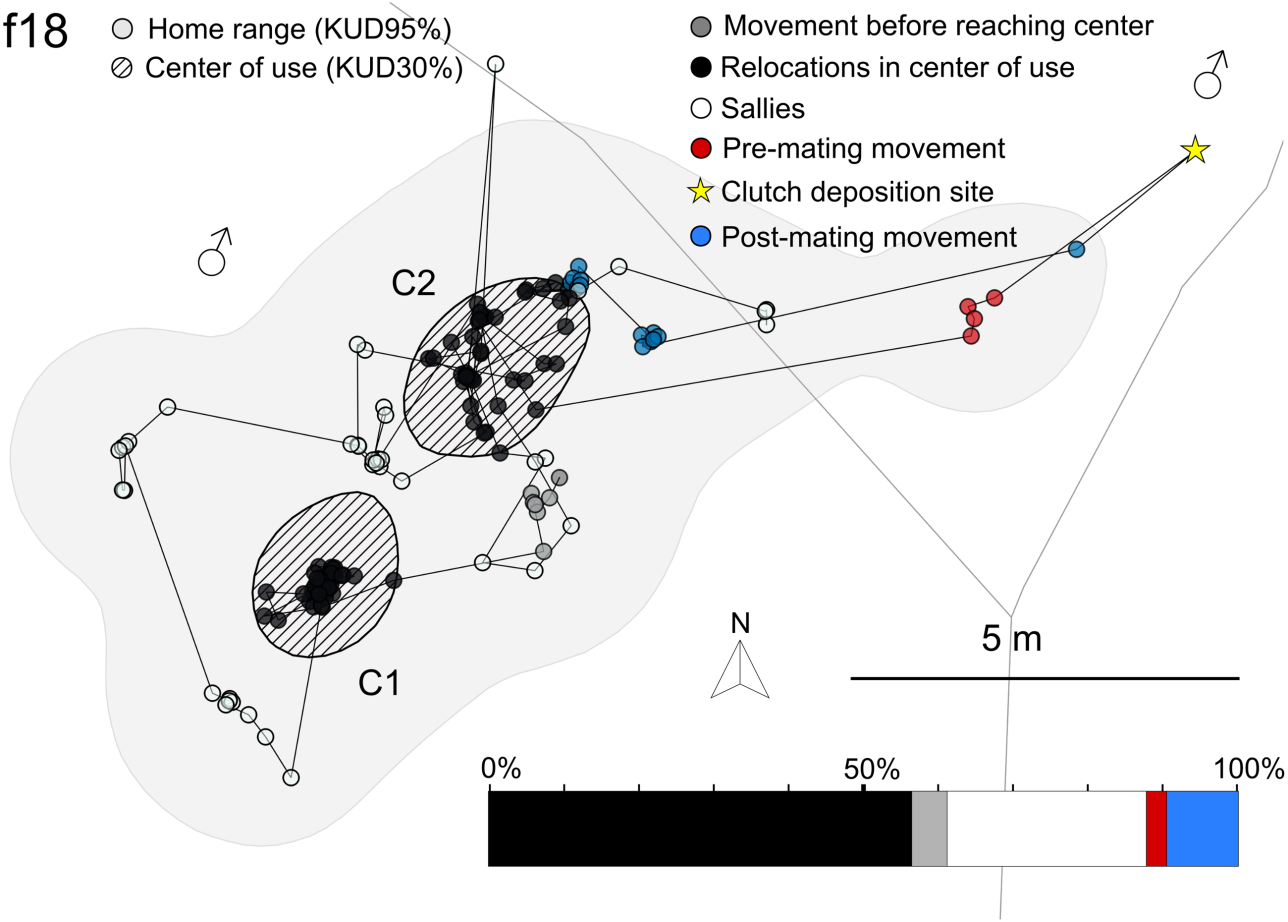
Female trajectory. Representative example (f18) of female trajectory with two centers of use. Centers of use (C1, C2) are striped (KUD30). HR area (KUD95) is shaded light grey. Relocalization points after tagging before reaching a center of use are shown in dark grey. Datapoints in the center of use are indicated in black, sallies to the surrounding are hollow, pre-mating movement is indicated with red and post mating movement until the next center of use is reached with blue dots. The same color code was used for the bar chart displaying the female’s time management. The oviposition site is indicated by a yellow star. Territories of surrounding males were estimated with the Voronoi approach and marked with a marssymbol.

Females spent over 60% of their time in their centers of use, ∼20% on sallies to the surrounding area and ∼10% in courtship and mating (Table 1, Fig. S18).

### Influence of density of surrounding males on female spatial characteristics

Neither size nor number of the female centers of use was correlated to the mean density of males in their surrounding (KUD30 size: linear regression: *n* = 17, SE = 13.9, slope = −2.3, R^2^ = −0.035, *p* = 0.51; number of centers: Spearman correlation: *n* = 17, *S* = 753.7, rho = 0.08, *p* = 0.8). Similarly, we found no correlation between the mean male density and the percent of time that females spent in the center of use or on sallies (linear regression: time spent in centers of use: *n* = 17, SE = 13.6, slope = 0.37, *R*^2^ = 0.01, *p* = 0.28; percent of time spent on sallies: *n* = 17, SE = 14.1, slope = 0.06, R^2^ = −0.06, *p* = 0.86).

### Approach of previous egg deposition site

Although no female was observed to attend a clutch, six out of 11 females were found in proximity (<2.5 m) to their first clutch again during tracking. Females revisited the area after different time periods (min = 4 d 23 h, max = 11 d 8 h) but mostly (five out of six) during another courtship/mating. Three out of six females passed by the previous clutch during the next courtship before mating with the same male again. Another female visited the first deposition site on a sally that was later followed by a courtship. One female who chose a different male for the second mating detoured past the previous oviposition site on her way back to a center of use after mating, a second female revisited the first egg deposition site during a sally.

Clutches from the same partner were on average 1.8 m apart (*n* = 3, mean = 1.8 m, range = 0.8–3.1 m), while clutches with different males were on average 8.7 m apart (*n* = 2, mean = 8.7 m, range = 7.6–9.8 m).

### Are tracking data representative for female long-term home range?

**Figure 6:**
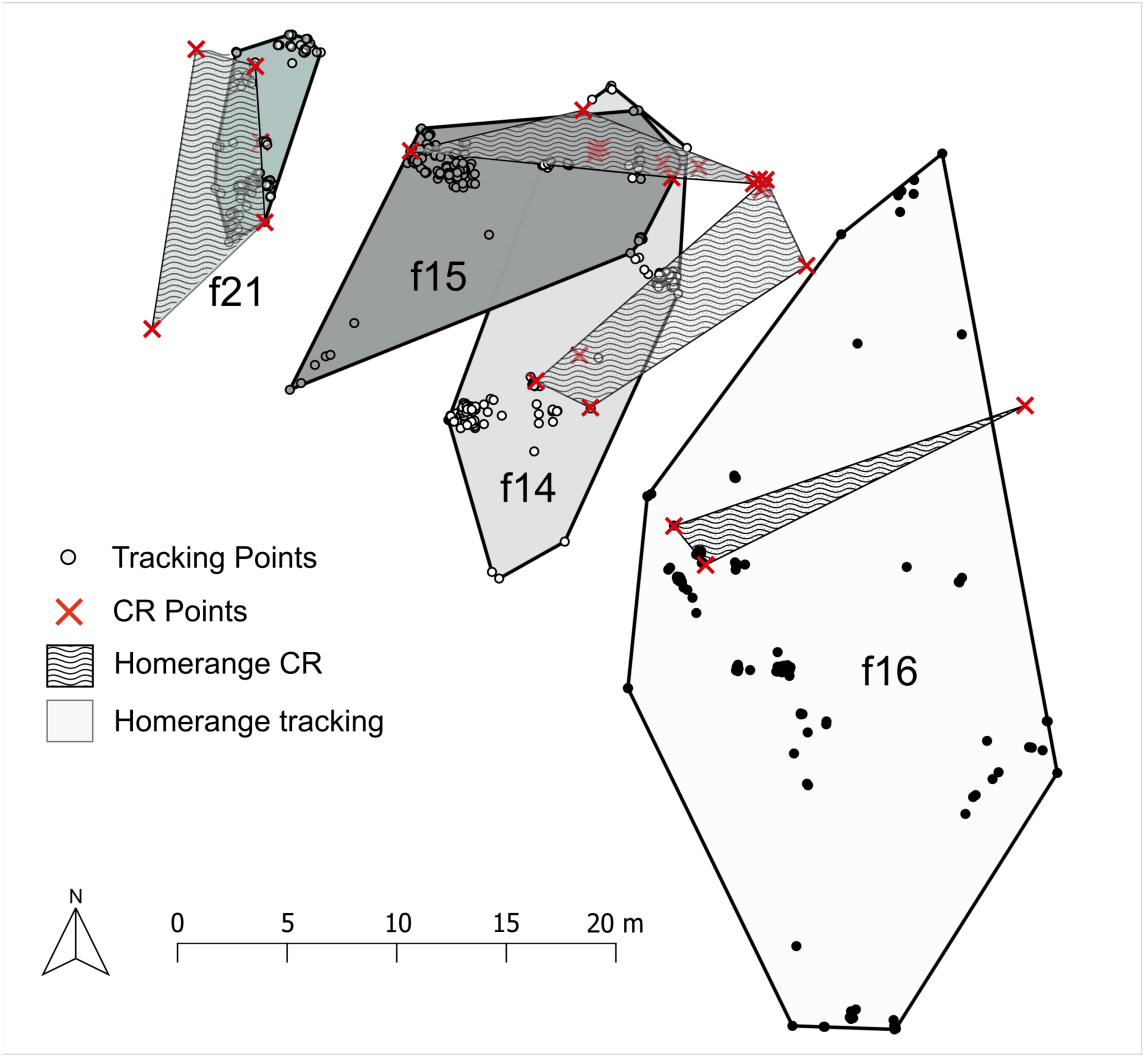
Long term site fidelity represented with short term tracking. MCP areas calculated from tracking and CMR dataset are shown for four females. MCP100 Polygons for the respective female from the two datasets always overlapped. CMR capture points are marked with a red cross, tracking points are shown as dots. Tracking MCP100 polygons are shown in plane colors (white, light grey, dark grey, green-grey) while corresponding CMR MCP100 are shown in the same color but with wavy fill.

MCP100 polygons created from the CMR dataset (time span: between 24 January and 14 April) and tracking dataset (up to 17 days) always overlapped (*n* = 9, mean = 46.8 ± 41.27%, range = 8.7–136.5%) (Fig. 6). For all but one female the MCP100 area was smaller when calculated from the CMR dataset (Table S4). The same pattern was found when comparing average MCP100 areas (CMR: *n* = 23, mean = 74.7 ± 108.9 m^2^, range = 1.7–436.7 m^2^; tracking: *n* = 17, mean = 138.7  ± 129 m^2^, range = 9.9–485.7 m^2^). The mean tracking MCP100 HR was on average 1.9 times larger than HR areas calculated from the mean CMR MCP100 HR.

### Behavioral observations

Females equipped with a tag performed all key behaviors described for untagged female *A. femoralis* (Table S1). We observed feeding on six occasions. In line with several studies (e.g., Ringler, Ursprung & Hödl, 2009; Roithmair, 1992; Roithmair, 1994) no female engaged in any agonistic interaction. One female was observed transporting a tadpole. We observed a total of 21 courtship events involving 12 tagged females (Fig. S19). For seven females, we observed two courtships during tracking. The average time between two successive ovipositions by the same female was eight days (*n* = 7; SD = ±2,4 days; min = 5 days; max = 12 days) and the average clutch size was 17.4 eggs (*n* = 11; SD = ±3,8; min = 11; max = 25) confirming observations for captive *A. femoralis* (Weygoldt, 1980). Out of seven females who were observed in two successful courtships during tracking, three mated with the same male twice, three chose a different male for the second mating event and on one occasion, the second male could not be captured and identified after clutch deposition.

## Discussion

We observed different activity patterns depending on the reproductive state of the females (Fig. 2). Movement was generally low but increased significantly in the afternoons of courtship initiation days. The timing coincided with the peak in male calling activity (Kaefer et al., 2012); Ringer et al., pers. obs., 2018), suggesting that acoustic cues facilitate mate-directed movement (Gerhardt, 1991; Sinsch, 2010). Females left the oviposition site in the morning when male calling activity was low, indicating that other factors than male vocalization influenced the decision where to go after mating.

In line with Bellis (1962) and Beck et al. (2017), we found a significant correlation of the daily movement with both temperature and cumulative rainfall (Fig. S1). Frogs moved more on days with lower temperatures, reflecting the fact that environmental temperatures exceeding the optimal range of ectotherms affect their physiological function and influence their behavioral performance (Bellis, 1962; Navas, 1996; Navas, Gomes & Carvalho, 2008; Beck et al., 2017). As habitat temperatures in the tropics are close to the upper thermal limits of amphibians, thermal tolerances of tropical species are narrow, making frogs vulnerable to climatic fluctuations (Duarte et al., 2011; Bonetti & Wiens, 2014; Sunday et al., 2014). Although cumulative rainfall was significantly correlated with female movement, the respective regression slope of 0.02 indicates a weak effect, as previously reported for our study species (Beck et al., 2017). However, rainfall is a strong predictor of male calling activity on a seasonal scale (Kaefer et al., 2012) and male vocal signals have in turn been found to stimulate estrogen changes in females of many anuran species (for review see Wilczynski & Lynch, 2011). Therefore, rainfall likely influences the timing of female movement also indirectly via increased male calling activity.

Increased travel distances on days with courtship and mating suggest that reproductive behavior is an important factor in prompting female movement (Figs. 3A, 3B). Between matings, females spent most of their time in a few smaller centers and we found no evidence for further goal-directed movement such as foraging excursions, as observed in *Dendrobates auratus* (Pough & Taigen, 1990).

Even though females repeatedly left their centers of use (Fig. S18), wide-ranging exploratory behavior (described for *Oophaga pumilio* females (Brust, 1990) and *D. auratus* (Summers, 1989) but not *A. femoralis* males (Beck et al., 2017) was not observed in *A. femoralis* females. A comparison with long-term CMR data of the study population demonstrated site fidelity of females beyond tracking periods, confirming reports on long-term site fidelity in this species (Ringler, Ursprung & Hödl, 2009). Additionally, females showed homing behavior after transporting tadpoles that were experimentally placed on their back (Pašukonis et al., 2017), suggesting benefits of returning to a known site. Females could, for example, profit from experience with a mating partner (Rosenthal, 2017) or enhanced larval survival rate by taking over tadpole transport if the male disappears, which has been demonstrated in *A. femoralis* (Silverstone, 1976; Ringler et al., 2013; Ringler et al., 2015).

Even though we never witnessed clutch attendance, all females were close to their first clutch during or after the following mating event, allowing a survey of older clutches or respective mating partners. Half of the females who were observed in two courtships mated with the same male twice, despite the presence of other neighbors. Preference for one mating partner might facilitate inspection of previous clutches during following courtships without additional risk or energy investments. While female mate-choice was suggested to be non-selective on a seasonal scale in *A. femoralis* (Ursprung et al., 2011) and in the poison frog *O. pumilio* (Meuche et al., 2013), temporary non-random mate choice in our study population is further supported by observations of females occasionally ignoring a close male before traveling longer distances to their previous partner for another mating.

The movement extent observed for *A. femoralis* females was far below the reported measures for invasive or migratory species (e.g., annual range expansion of up to 55 km for *Rhinella marina* (Phillips et al., 2007), migration distances of up to 15 km for *Rana lessonae* (Tunner & Karpati, 1997) and up to 4 km for *Epidalea calamita* (Sinsch et al., 2012)). Likewise, female average home ranges (MCP: 107 m^2^) were smaller than reported for migratory anurans (e.g., between 100 and 1,000 m^2^ e.g., in *Rana sylvatica* (Rittenhouse & Semlitsch, 2007) and *Lithobates pipiens* (Swanson et al., 2018) and up to 1,900 m^2^ in *Bufo bufo* (Sinsch, 1987); for reviews see Pittman, Osbourn & Semlitsch, 2014; Brown, Morales & Summers, 2010; Joly, 2019) .

Intra-specific differences in female space use (Table S3) likely originate from population densities, long-term site choice and the availability and quality of resources around the centers of use. For example, temporary resources like pools, spawning sites, conspecifics or food sources were described to influence movements of poison frogs (pool availability: *A. paleovarzensis*: Rocha, Lima & Kaefer, 2018; phytotelmata availability: *O. pumilio*: Donnelly, 1989; Pröhl & Berke, 2001; *Ranitomeya ventrimaculata*: Poelman & Dicke, 2008; conspecific attraction: *O. pumilio*: Folt, Donnelly & Guyer, 2018; momentary food resources: *A. femoralis*: M Ringler, 2016–2019, in preparation). In our study, female time management and space use was not correlated to the number of surrounding males, suggesting that females adjust their fine-scale movement to other factors but male density, such as the immediate behavior or location of surrounding frogs (e.g., Ward, Webster & Hart, 2006).

Species differences in HR size are likely to arise from different mating systems and parental care strategies. For example, the spatial behavior of *R. imitator* is dictated by monogamous pair bonding and biparental care. Both sexes mutually defend a territory of ∼5 m^2^ and care for their young, with no differences in space use between male and female individuals (Symula, Schulte & Summers, 2001; Brown, Morales & Summers, 2008; Brown, Morales & Summers, 2009). In contrast, closely related *R. amazonica* are promiscuous and display male uniparental care (Poelman & Dicke, 2007). Male frogs defend territories that are typically four times smaller than the area used by females (up to 39 m^2^) that need to search for mates because males are not advertising their presence acoustically (Poelman & Dicke, 2008). Space use characteristics of *A. femoralis* females are comparable to other territorial leaf-litter poison frogs with uniparental male care, where female investment is confined to oviposition (e.g., *Amereega trivittata* (Neu et al., 2016)). However, areas used by *A. femoralis* females exceed the HR reported for species that depend on phytotelma pools to rear their young (e.g., *R. variabilis* (Brown, Morales & Summers, 2009)) or feed their tadpoles (e.g., *O. pumilio* (Haase & Pröhl, 2002)).

Using different methods for data collection (e.g., CMR or tracking) or analysis (e.g., different definitions and algorithms for HR estimation) causes variations in resulting space use metrics. We tackled this issue by applying two HR estimators (Fig. 4) and comparing datasets from tracking and CMR. Although absolute size estimations between MCP and KUD differed in our study (Table S3), ecologically relevant patterns (e.g., the increase in HR size on days with courtship and mating) were consistently found with both analysis methods.

Contrary to CMR methods, tracking allows to locate specific individuals frequently, which is necessary for studies of fine-scale movement (Richards, Sinsch & Alford, 1994; Kays et al., 2015). Female *A. femoralis* are particularly challenging to survey via CMR as they do not engage in any visual or acoustic displays but can be found by localizing males that emit distinct courtship calls (Stückler et al., 2019). Therefore, reproduction associated capture points (29% of female captures in our dataset) are overrepresented in the CMR dataset and can bias female HR towards the locations of the mating partners. However, tracking with tags fixed on waistbands has several disadvantages: frogs become more conspicuous to predators and slower in escaping (Kenward, 1987), they need to be captured and manually checked for wounds and localizing them multiple times per day might disturb their behavior. While we did not investigate possible effects of tagging on feeding success, total movement or long-term reproductive success (Langkilde & Alford, 2002; Blomquist & Hunter, 2007; Rowley & Alford, 2007), equipping wild *A. femoralis* females with a tag did not alter their known behavioral repertoire including mating (Fig. S19), tadpole transport, and feeding.

## Conclusions

Fine-scale movement of sedentary amphibians is understudied, though critical to determine factors influencing the decision to move (e.g., this study), spatial memory and navigational abilities (e.g., Pašukonis et al., 2016) or to develop mechanistic movement models (e.g., McClintock et al., 2012). Using tracking, we provide the first fine-scale metrics of the space use patterns and time budget of sedentary inconspicuous female poison frogs. Our study demonstrates how short-term tracking can be used to refine information gathered by a conventional CMR approach, filling the knowledge gap about patterns, causation and function of fine-scale spatio-temporal behavior in amphibians. Future studies spanning multiple genera with distinct caretaking strategies could address how mating systems, parental duties and dependence on reproductive resources drive species and sex differences in fine-scale spatial behavior in this clade.

##  Supplemental Information

10.7717/peerj.8920/supp-1 Table S1 Characteristics and key behaviors of female *A. femoralis* in natural populations The following characteristics and behaviors were described for wild *A. femoralis* females and serve as qualitative reference for tagged females.

10.7717/peerj.8920/supp-2 Table S2 Correlation of tracking time and home range size Results of linear regression of different HR estimates with tracking time are shown. The correlations were not significant if only female *A. femoralis* tracked for 14 days or longer were included in the analysis. Significant p-values are given in bold.

10.7717/peerj.8920/supp-3 Table S3 Home range comparison after 14 days Estimated HR for nine female *A. femoralis* after 14 days of continuous tracking are reported, the number of reproductive events at this time is indicated as ‘courtship’. Minima and maxima are given in bold.

10.7717/peerj.8920/supp-4 Table S4 Home range estimations from different datasets*.* Only females with more than four capture point in the CR dataset were included into the analysis. HR for CR and tracking dataset was calculated with MCP method using all datapoints (MCP100). Shapefiles were intersected and the overlap of the polygons was calculated (shown as % of the mostly larger MCP100 area). Tracking duration, number of tracking points and time span of CR data collection (first to last capture point) are shown for each female.

10.7717/peerj.8920/supp-5 Figure S1 Environmental factors influencing movement The correlation between the moved daily distances and the environmental factors **(A)** ‘temperature’ and **(B)**‘rainfall’ is shown. We found a significant correlation with both variables. While temperature was negatively correlated with movement of *A. femoralis* females (Spearman’s rho = −0.35, *p* = 0.001), rainfall showed a positive correlation (Spearman’s rho = 0.36, *p* = 0.002), as represented by the linear regression lines.

10.7717/peerj.8920/supp-6 Figure S2 Home range in relation to reproductive behavior The development of the home range (MCP95) per tracking day is shown for 6 females tracked for at least 16 days **(A–F)**. Grey bars indicate reproductive events (courtship-mating) which typically span two days. Home range estimations using KUD95 showed similar patterns. Most females showed the highest increase in home range size on the days of courtship and mating.

10.7717/peerj.8920/supp-7 Figure S3 Trajectory of female f03 Female trajectory with two centers of use. Centers of use (C1, C2) are striped (KUD30). HR area (KUD95) is shaded light grey. Relocalization points after tagging before reaching a center of use are shown in dark grey. Datapoints in the center of use are indicated in black, sallies to the surrounding are marked with hollow dots, pre-mating movement is indicated with red and post-mating movement until the next center of use is reached with blue dots. The egg deposition site is indicated by a yellow star. Territories of surrounding males were estimated with the Voronoi approach and marked with a marssymbol. This female was tracked for 15 days.

10.7717/peerj.8920/supp-8 Figure S4 Trajectory of female f05 Female trajectory with one center of use. The center of use is striped (KUD30). HR area (KUD95) is shaded light grey. Relocalization points after tagging before reaching a center of use are shown in dark grey. Datapoints in the center of use are indicated in black, sallies to the surrounding are marked with hollow dots, pre-mating movement is indicated with red and post-mating movement until the next center of use is reached with blue dots. The egg deposition site is indicated by a yellow star. Territories of surrounding males were estimated with the Voronoi approach and marked with a marssymbol. This female was tracked for 16 days.

10.7717/peerj.8920/supp-9 Figure S5 Trajectory of female f06 Female trajectory with one center of use. The center of use is striped (KUD30). HR area (KUD95) is shaded light grey. Relocalization points after tagging before reaching a center of use are shown in dark grey. Datapoints in the center of use are indicated in black, sallies to the surrounding are marked with hollow dots, pre-mating movement is indicated with red and post-mating movement until the next center of use is reached with blue dots. The egg deposition site is indicated by a yellow star. This female was observed transporting one tadpole, as indicated with green datapoints. Territories of surrounding males were estimated with the Voronoi approach and marked with a marssymbol. This female was tracked for 16 days.

10.7717/peerj.8920/supp-10 Figure S6 Trajectory of female f08 Female trajectory with one center of use. The center of use is striped (KUD30). HR area (KUD95) is shaded light grey. Relocalization points after tagging before reaching a center of use are shown in dark grey. Datapoints in the center of use are indicated in black, sallies to the surrounding are marked with hollow dots. No courtship/mating event was observed for this female. Territories of surrounding males were estimated with the Voronoi approach and marked with a marssymbol. This female was tracked for 7 days.

10.7717/peerj.8920/supp-11 Figure S7 Trajectory of female f09 Female trajectory with one center of use. The center of use is striped (KUD30). HR area (KUD95) is shaded light grey. Relocalization points after tagging before reaching a center of use are shown in dark grey. Datapoints in the center of use are indicated in black, sallies to the surrounding are marked with hollow dots. No courtship/mating event was observed for this female. Territories of surrounding males were estimated with the Voronoi approach and marked with a marssymbol. This female was tracked for six days.

10.7717/peerj.8920/supp-12 Figure S12 Trajectory of female f10 Female trajectory with one center of use. The center of use is striped (KUD30). HR area (KUD95) is shaded light grey. Relocalization points after tagging before reaching a center of use are shown in dark grey. Datapoints in the center of use are indicated in black, sallies to the surrounding are marked with hollow dots. No courtship/mating event was observed for this female. Territories of surrounding males were estimated with the Voronoi approach and marked with a marssymbol. This female was tracked for 13 days.

10.7717/peerj.8920/supp-13 Figure S9 Trajectory of female f12 Female trajectory with three centers of use. Centers of use (C1, C2, C3) are striped (KUD30). HR area (KUD95) is shaded light grey. Datapoints in the center of use are indicated in black, sallies to the surrounding are marked with hollow dots, pre-mating movement is indicated with red and post-mating movement until the next center of use is reached with blue dots. The egg deposition site is indicated by a yellow star. Territories of surrounding males were estimated with the Voronoi approach and marked with a marssymbol. This female was tracked for ten days.

10.7717/peerj.8920/supp-14 Figure S10 Trajectory of female f13 Female trajectory with three centers of use. Centers of use (C1, C2, C3) are striped (KUD30). HR area (KUD95) is shaded light grey. Datapoints in the center of use are indicated in black, sallies to the surrounding are marked with hollow dots, pre-mating movement is indicated with red and post-mating movement until the next center of use is reached with blue dots. The egg deposition site is indicated by a yellow star. This female was tagged after oviposition. Territories of surrounding males were estimated with the Voronoi approach and marked with a marssymbol. This female was tracked for eight days.

10.7717/peerj.8920/supp-15 Figure S11 Trajectory of female f14 Female trajectory with two centers of use. Centers of use (C1, C2) are striped (KUD30). HR area (KUD95) is shaded light grey. Relocalization points after tagging before reaching a center of use are shown in dark grey. Datapoints in the center of use are indicated in black, sallies to the surrounding are marked with hollow dots, pre-mating movement is indicated with red and post-mating movement until the next center of use is reached with blue dots. The mating site is indicated by a yellow point. This female was detagged after we observed two consecutive courtship/mating events without successful clutch deposition. Territories of surrounding males were estimated with the Voronoi approach and marked with a marssymbol. This female was tracked for 17 days.

10.7717/peerj.8920/supp-16 Figure S12 Trajectory of female f16 Female trajectory with one center of use. The center of use is striped (KUD30). HR area (KUD95) is shaded light grey. Relocalization points after tagging before reaching a center of use are shown in dark grey. Datapoints in the center of use are indicated in black, sallies to the surrounding are marked with hollow dots, pre-mating movement is indicated with red and post-mating movement until the next center of use is reached with blue dots. The egg deposition site is indicated by a yellow star. Territories of surrounding males were estimated with the Voronoi approach and marked with a marssymbol. This female was tracked for 17 days.

10.7717/peerj.8920/supp-17 Figure S13 Trajectory of female f17 Female trajectory with one center of use. The center of use is striped (KUD30). HR area (KUD95) is shaded light grey. Relocalization points after tagging before reaching a center of use are shown in dark grey. Datapoints in the center of use are indicated in black, sallies to the surrounding are marked with hollow dots. No courtship/mating event was observed for this female. Territories of surrounding males were estimated with the Voronoi approach and marked with a marssymbol. This female was tracked for seven days.

10.7717/peerj.8920/supp-18 Figure S14 Trajectory of female f19 Female trajectory with one center of use. The center of use is striped (KUD30). HR area (KUD95) is shaded light grey. Relocalization points after tagging before reaching a center of use are shown in dark grey. Datapoints in the center of use are indicated in black. No sallies or courtship/mating events were observed for this female. Territories of surrounding males were estimated with the Voronoi approach and marked with a marssymbol. This female was tracked for ten days.

10.7717/peerj.8920/supp-19 Figure S15 Trajectory of female f20 Female trajectory with one center of use. The center of use is striped (KUD30). HR area (KUD95) is shaded light grey. Datapoints in the center of use are indicated in black, sallies to the surrounding are marked with hollow dots, pre-mating movement is indicated with red and post-mating movement until the next center of use is reached with blue dots. The egg deposition site is indicated by a yellow star. Territories of surrounding males were estimated with the Voronoi approach and marked with a marssymbol. This female was tracked for 17 days.

10.7717/peerj.8920/supp-20 Figure S16 Trajectory of female f21 Female trajectory with one center of use. The center of use is striped (KUD30). HR area (KUD95) is shaded light grey. Relocalization points after tagging before reaching a center of use are shown in dark grey. Datapoints in the center of use are indicated in black, sallies to the surrounding are marked with hollow dots, pre-mating movement is indicated with red and post-mating movement until the next center of use is reached with blue dots. The egg deposition site is indicated by a yellow star. Territories of surrounding males were estimated with the Voronoi approach and marked with a marssymbol. This female was tracked for 14 days.

10.7717/peerj.8920/supp-21 Figure S17 Trajectory of female f22 Female trajectory with one center of use. The center of use is striped (KUD30). HR area (KUD95) is shaded light grey. Relocalization points after tagging before reaching a center of use are shown in dark grey. Datapoints in the center of use are indicated in black, sallies to the surrounding are marked with hollow dots, pre-mating movement is indicated with red and post-mating movement until the next center of use is reached with blue dots. The egg deposition site is indicated by a yellow star. Territories of surrounding males were estimated with the Voronoi approach and marked with a marssymbol. This female was tracked for ten days.

10.7717/peerj.8920/supp-22 Figure S18 Female time budget The percentage of time spent (i) on movement after tagging before reaching a center of use (grey), (ii) in the center of use (black), (iii) on sallies outside of the center (white), (iv) on pre-mating movement towards a mating partner (red) and (v) on post-mating movement until reaching the next center of use (blue) is illustrated.

10.7717/peerj.8920/supp-23 Figure S19 Reproduction-related behavior of tagged females*.* Female *A. femoralis* equipped with a tag (right side) attending a calling male (left side) after actively approaching him. Photo credit: Andrius Pašukonis.

10.7717/peerj.8920/supp-24 Data S1 Raw Data: tracking Spatial coordinates of tracked females

10.7717/peerj.8920/supp-25 Data S2 Raw Data CMR CMR dataset of all frogs in 2016 for access in ArcGIS (files: APL, CPG, DBF, PRJ, SBN, SBX, SHP, XML, SHX): ID, sex, date and time of relocations as well as the frogs’ activity at that time.

## Additional Information and Declarations

### Field Study Permissions

The following information was supplied relating to field study approvals (i.e., approving body and any reference numbers): The relevant permission to conduct fieldwork was provided by the prefect of the Guiana region (Arrete no 2011-44 /DEAL/SMNBSP/BSP du 19/07/2011). In addition to this permit, all protocols for fieldwork were approved by the scientific committee of the station Saut Pararé (managed by the Centre Nationale de la Recherche scientific, CNRS). The decision of the scientific committee was communicated as oral agreement by the technical director of the station, Philippe Gaucher (representing CNRS) to Eva and Max Ringler (representing the University of Vienna).

### Data Availability

The following information was supplied regarding data availability: The spatial raw data from tracking are available in the Supplementary Files.
