# Supplementary material for: Reproductive behavior drives female space use in a sedentary Neotropical frog
Source: PeerJ. 2020 Apr 17;8:e8920. doi: 10.7717/peerj.8920 (PMC7169969; doi:10.7717/peerj.8920)
Supplement: Table S1 — The following characteristics and behaviors were described for wild A. femoralis females and serve as qualitative reference for tagged females. [file peerj-08-8920-s001.docx]

**Supplementary Table S1:**

**Characteristics and key behaviors of female A. femoralis in natural populations.**

The following characteristics and behaviors were described for wild A. femoralis females and serve as qualitative reference for tagged females.

| **Category** | **Characteristics & behaviors** | **Reference literature** | **Description** |
| --- | --- | --- | --- |
| *General* | Feeding | (Parmelee, 1999) | Food quality: orthopterans, coleopterans, adult arthropods, ants /mites, larvae |
|  | Site fidelity | (Ringler et al., 2009) | Intra-annual female site fidelity |
| *Reproduction* | Temporal patterns of courtship | (Stückler et al., 2019) | Courtship starts in the afternoon ~17h and continues on the next morning, ending in mating |
|  | Amplexus | (Stückler et al., 2019) | Cephalic amplexus |
|  | Oviposition | (Stückler et al., 2019) | Oviposition in the morning, ~8h |
|  | Discontinued courtship | (Stückler et al., 2019) | 2 out of 29 females discontinued the courtship |
| *Social* | Agonistic interactions with females or males | (Ringler et al., 2009; Roithmair, 1992, 1994) | No agonistic interactions observed |
| *Parental care* | Tadpole transport | (Ringler, et al., 2015; Silverstone, 1976) | Female compensate for absent male and shuttle tadpoles (7.8 % in the field) |
| *Mortality* | Predation | (Beck et al., 2017; Pašukonis et al., 2014; Ringler, Ursprung, & Hödl, 2010) | Snakes and spiders described as predators |
|  | Survival rate | (Ringler et al., 2009) | Inter-annual survival < 20 % |

# References

Beck KB, Loretto M-C, Ringler M, Hödl W, Pašukonis A. 2017. Relying on known or exploring for new? Movement patterns and reproductive resource use in a tadpole-transporting frog. *PeerJ*:e3745. https://doi.org/10.7717/peerj.3745

Parmelee JR. 1999. Trophic ecology of a tropical anuran assemblage. *Scientific Papers, Natural History Museum, The University of Kansas*, 11:1–59. https://doi.org/http://dx.doi.org/10.5962/bhl.title.16167

Pašukonis A, Warrington I, Ringler M, Hödl W. 2014. Poison frogs rely on experience to find the way home in the rainforest. *Biology Letters*, 10:20140642. https://doi.org/http://dx.doi.org/10.1098/rsbl.2014.0642

Ringler M, Ursprung E, Hödl W. 2009. Site fidelity and patterns of short- and long-term movement in the brilliant-thighed poison frog Allobates femoralis ( Aromobatidae ). *Behavioral Ecology and Sociobiology*, 63:1281–1293. https://doi.org/10.1007/s00265-009-0793-7

Ringler M, Ursprung E, Hödl W. 2010. Predation on Allobates femoralis by the colubrid snake Xenopholis scalaris. *Herpetology Notes*, 3:301–304.

Ringler E, Pašukonis A, Fitch WT, Huber L, Hödl W, Ringler M. 2015. Flexible compensation of uniparental care: Female poison frogs take over when males disappear. *Behavioral Ecology*, 26:1219–1225. https://doi.org/10.1093/beheco/arv069

Roithmair ME. 1992. Territoriality and male mating success in the dart-poison frog, Epipedobates femoralis (Dendrobatidae, Anura). *Ethology*, 92:331–343.

Roithmair ME. 1994. Field studies on reproductive behavior in two Dart-Poison Frog species (Epipedobates femoralis, Epipedobates trivittatus) in Amazonian Peru. *Herpetological Journal*, 4:77–85.

Silverstone PA. 1976. A revision of the poison-arrow frogs of the genus Phyllobates Bibron in Sagra (family Dendrobatidae). *Natural History Museum of Los Angeles County, Science Bulletin*, 27:1–58.

Stückler S, Ringler M, Pašukonis A, Weinlein S, Hödl W, Ringler E. 2019. Spatio-temporal Characteristics of the prolonged Courtship in the Brilliant-thighed Poison Frog, Allobates femoralis. *Herpetologica,* 75:268-279. https://doi.org/10.1655/Herpetologica-D-19-00010.1
