# Supplementary material for: Reproductive behavior drives female space use in a sedentary Neotropical frog
Source: PeerJ. 2020 Apr 17;8:e8920. doi: 10.7717/peerj.8920 (PMC7169969; doi:10.7717/peerj.8920)
Supplement: Table S2 — Results of linear regression of different HR estimates with tracking time are shown. The correlations were not significant if only female A. femoralis tracked for 14 days or longer were included in the analysis. Significant p-values are given in bold. [file peerj-08-8920-s002.docx]

**Supplementary Table S1:**

**Correlations of tracking time and home range size.**

Results of linear regression of different HR estimates with tracking time are shown. The correlations were not significant if only female A. femoralis tracked for 14 days or longer were included in the analysis. Significant p-values are given in bold.

| **Correlation with tracking time** | **Estimate** | **Linear regression** | **Significance** |  |
| --- | --- | --- | --- | --- |
| *HR complete dataset*  (n = 17) | MCP95 | slope = 19.93  R^2^ = 0.36 | ***p*** **= 0.006** |  |
| *HR complete dataset*  (n = 17) | KUD95 | slope = 22.7  R^2^ = 0.27 | ***p* = 0.019** |  |
| *Centers of use*  (n = 17) | KUD30 | slope = 18.6  R^2^ = 0.18 | ***p* = 0.05** |  |
| *HR ≥ 14 tracking days*  (n = 9) | MCP95 | slope = 2.39  R^2^ = - 0.09 | *p* = 0.58 |  |
| *HR ≥ 14 tracking days*  (n = 9) | KUD95 | slope = 4.23  R^2^ = - 0.08 | *p* = 0.53 |  |
| *Centers of use ≥ 14 tracking days*  (n = 9) | KUD30 | slope = 1.80  R^2^ = - 0.12 | *p* = 0.72 |  |
