# Supplementary material for: Reproductive behavior drives female space use in a sedentary Neotropical frog
Source: PeerJ. 2020 Apr 17;8:e8920. doi: 10.7717/peerj.8920 (PMC7169969; doi:10.7717/peerj.8920)
Supplement: Table S3 — Estimated HR for nine female A. femoralis after 14 days of continuous tracking are reported, the number of reproductive events at this time is indicated as ‘courtship’. Minima and maxima are given in bold. [file peerj-08-8920-s003.docx]

**Supplementary Table S3:**

**Home range comparison after 14 days**.

Estimated HR for 9 female A. femoralis after 14 days of continuous tracking are reported, the number of reproductive events at this time is indicated as ‘Courtship’. Minima and maxima are indicated bold.

| **Individual** | **Courtship events** | **HR MCP95 (m^2^)** | **HR KernelUD95 (m^2^)** |
| --- | --- | --- | --- |
| f18 | 1 | **24.8** | 64.7 |
| f21 | 2 | 26.8 | **59.7** |
| f15 | 1 | 51.2 | 91.5 |
| f5 | 2 | 101.3 | 175.4 |
| f14 | 2 | 104.4 | 242.3 |
| f20 | 1 | 114.3 | 250.1 |
| f3 | 2 | 192.9 | 447.5 |
| f6 | 1 | 206.7 | 229.7 |
| f16 | 1 | **285.2** | **564.9** |
| Mean | - | 123.1 | 236.2 |
| Median | - | 104.4 | 229.7 |
| SE | - | 88.8 | 172.5 |
