# Supplementary material for: Reproductive behavior drives female space use in a sedentary Neotropical frog
Source: PeerJ. 2020 Apr 17;8:e8920. doi: 10.7717/peerj.8920 (PMC7169969; doi:10.7717/peerj.8920)
Supplement: Table S4 — Only females with more than four capture point in the CR dataset were included into the analysis. HR for CR and tracking dataset was calculated with MCP method using all datapoints (MCP100). Shapefiles were intersected and the overlap of the polygons was calculated (shown as % of the mostly larger MCP100 area). Tracking duration, number of tracking points and time span of CR data collection (first to last capture point) are shown for each female. [file peerj-08-8920-s004.docx]

**Supplementary Table S4:**

**Home range estimations from different datasets*.***

Only females with more than 4 capture point in the CR dataset were included into the analysis. HR for CR and tracking dataset was calculated with MCP method using all datapoints (MCP100). Shapefiles were intersected and the overlap of the polygons was calculated (shown as % of the mostly larger MCP100 area). Tracking duration, number of tracking points and time span of CR data collection (first to last capture point) are shown for each female.

| **RECCO_ID** | **CR points** | **MCP100 (m^2^) CR** | **MCP100 (m^2^) tracking** | **Tracking duration** | **% overlap** |
| --- | --- | --- | --- | --- | --- |
| f13 | 6; 68 days | 18.3 | 210.6 | 8 days | 8.7 |
| f12 | 6; 57 days | 12.0 | 86.2 | 10 days | 13.9 |
| f5 | 11; 65 days | 25.2 | 133.4 | 16 days | 18.9 |
| f15 | 10; 70 days | 21.8 | 111.6 | 17 days | 19.5 |
| f14 | 8; 71 days | 42.2 | 137.2 | 17 days | 30.8 |
| f6 | 5; 67 days | 160.9 | 316.7 | 16 days | 50.8 |
| f19 | 4; 37 days | 17.1 | 25.9 | 10 days | 66.2 |
| f8 | 4; 66 days | 7.6 | 9.9 | 7 days | 76.2 |
| f21 | 5; 42 days | 6.0 | 26.9 | 14 days | 136.5 |
