# Supplementary figures and images for: Reproductive behavior drives female space use in a sedentary Neotropical frog
Source: PeerJ. 2020 Apr 17;8:e8920. doi: 10.7717/peerj.8920 (PMC7169969; doi:10.7717/peerj.8920)

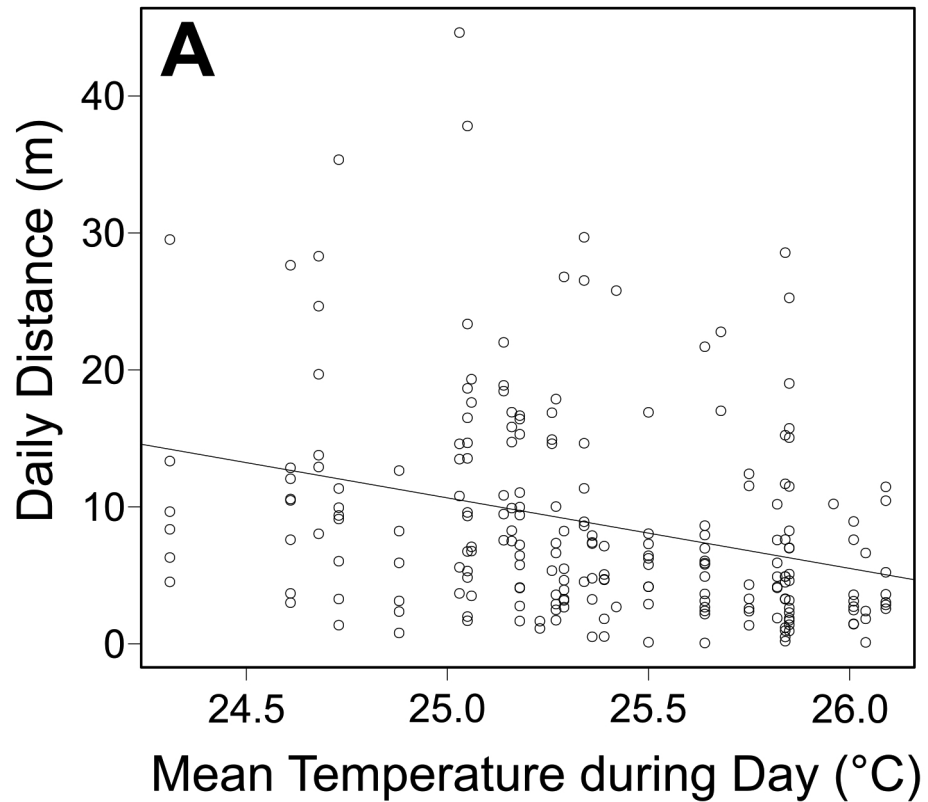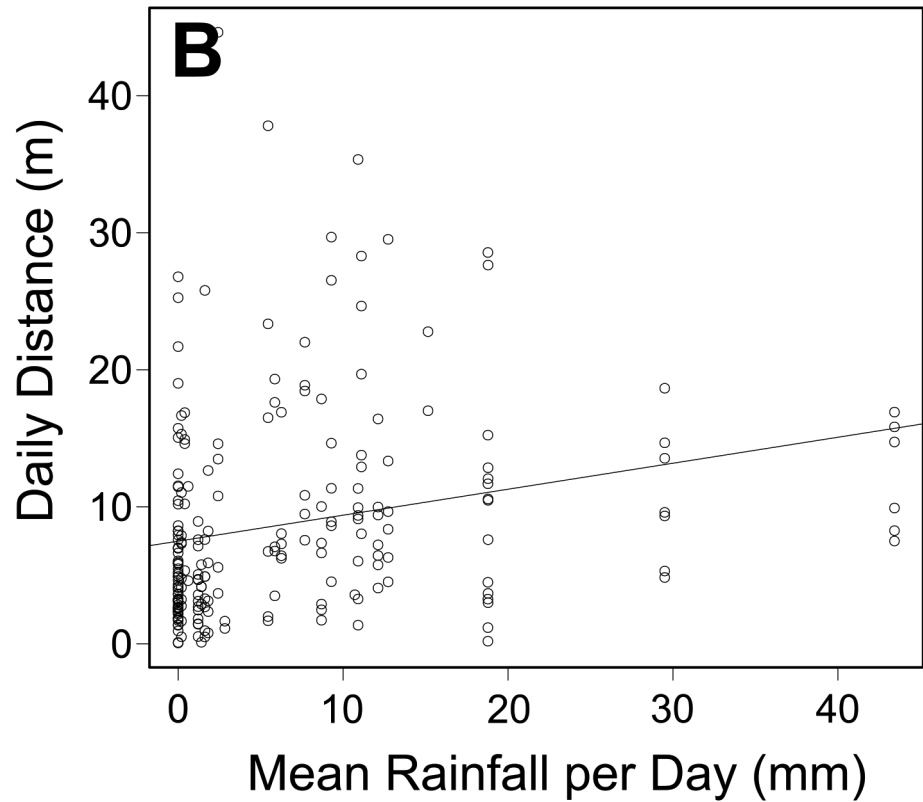

Supplement: Figure S1 — The correlation between the moved daily distances and the environmental factors (A) ‘temperature’ and (B)‘rainfall’ is shown. We found a significant correlation with both variables. While temperature was negatively correlated with movement of A. femoralis females (Spearman’s rho = −0.35, p = 0.001), rainfall showed a positive correlation (Spearman’s rho = 0.36, p = 0.002), as represented by the linear regression lines. [file peerj-08-8920-s005.pdf]

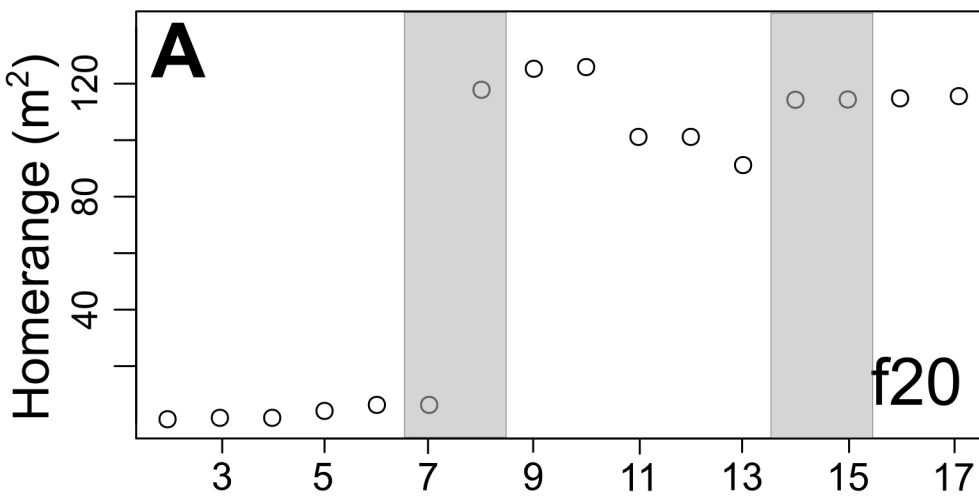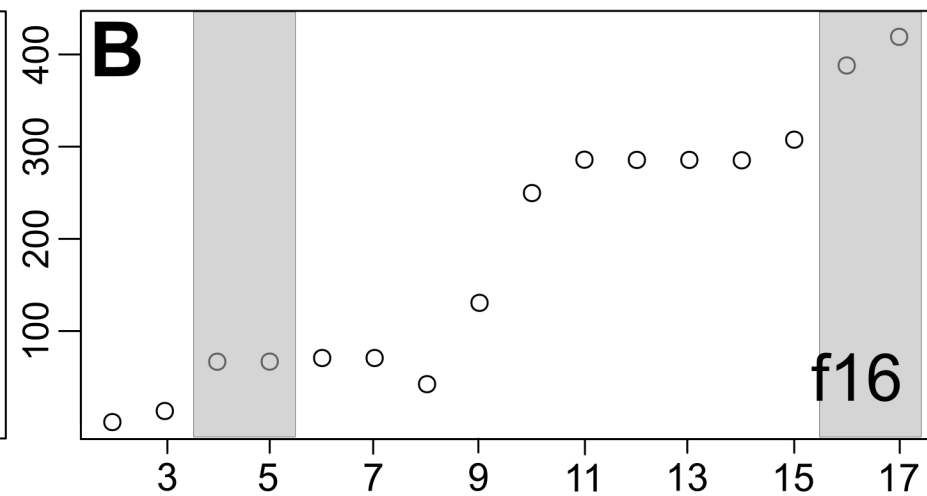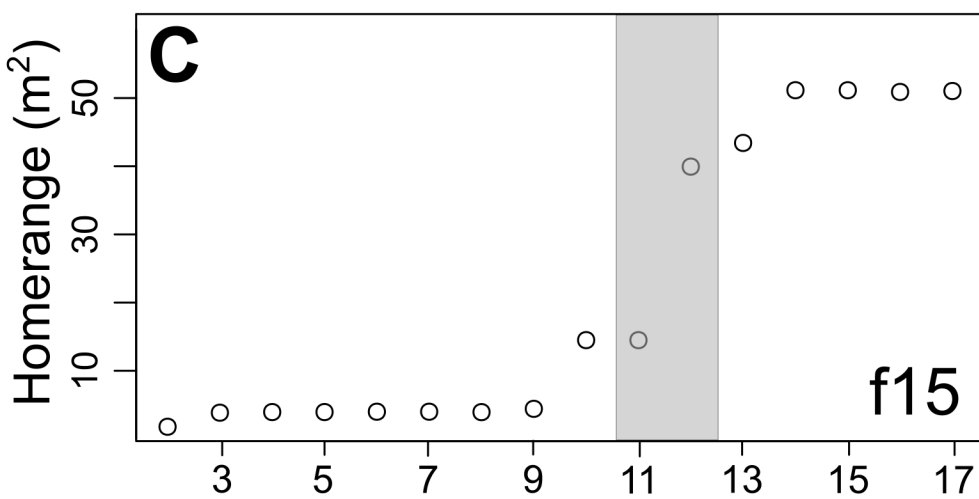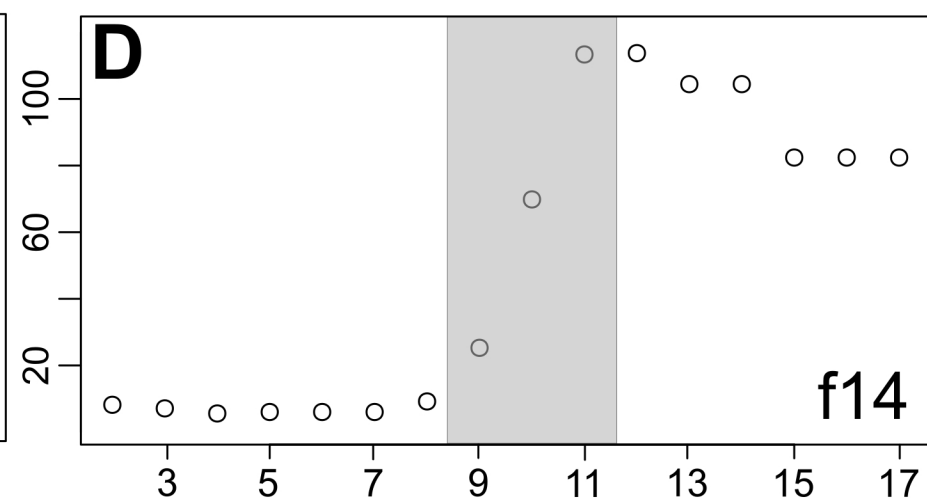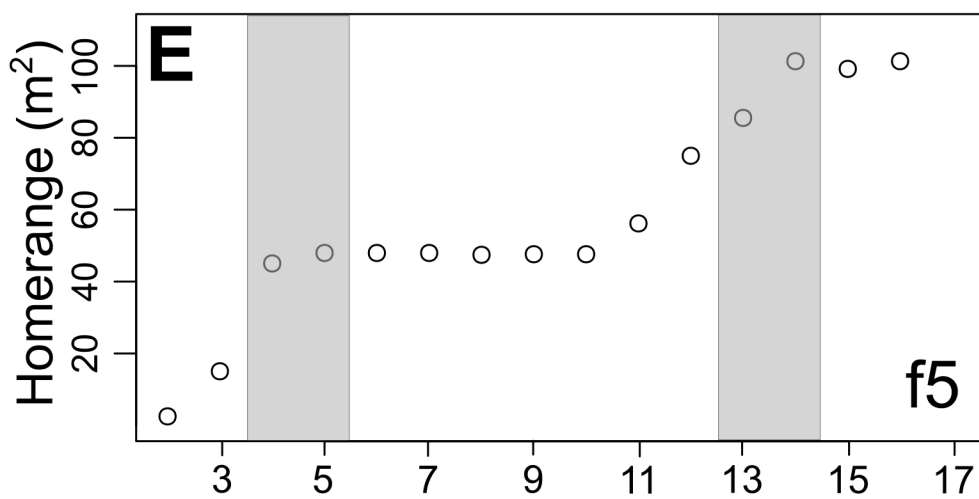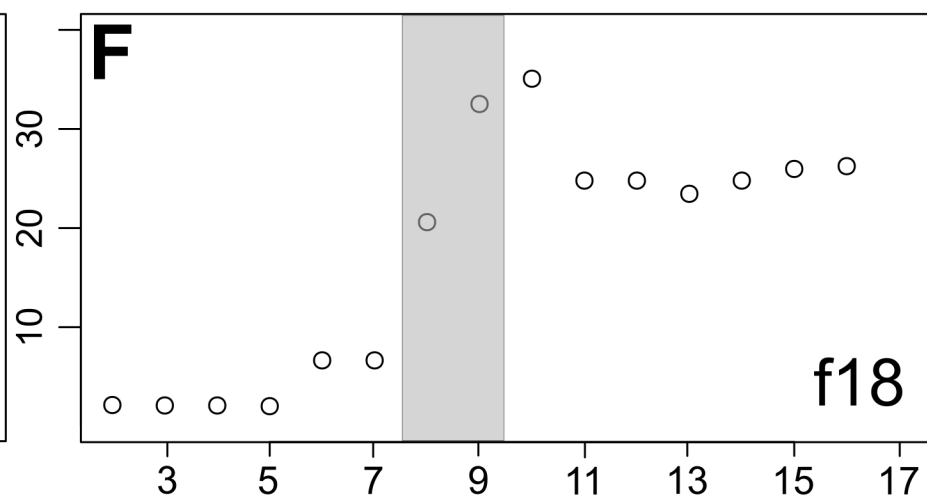

Tracking Days

Tracking Days

Supplement: Figure S2 — The development of the home range (MCP95) per tracking day is shown for 6 females tracked for at least 16 days (A–F). Grey bars indicate reproductive events (courtship-mating) which typically span two days. Home range estimations using KUD95 showed similar patterns. Most females showed the highest increase in home range size on the days of courtship and mating. [file peerj-08-8920-s006.pdf]

f03

○ Home range (KUD95%)

⊗ Center of use (KUD30%)

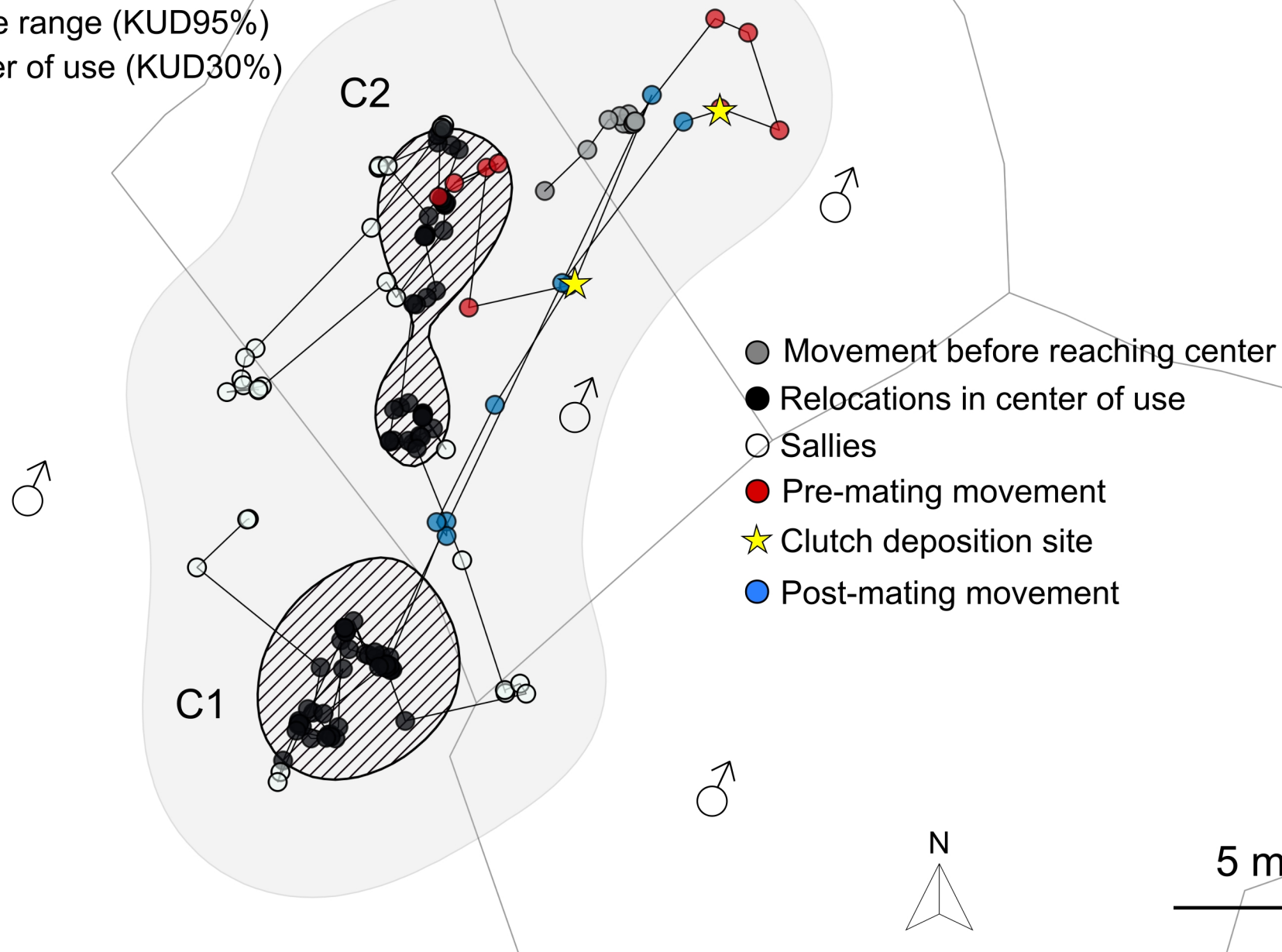

Supplement: Figure S3 — Female trajectory with two centers of use. Centers of use (C1, C2) are striped (KUD30). HR area (KUD95) is shaded light grey. Relocalization points after tagging before reaching a center of use are shown in dark grey. Datapoints in the center of use are indicated in black, sallies to the surrounding are marked with hollow dots, pre-mating movement is indicated with red and post-mating movement until the next center of use is reached with blue dots. The egg deposition site is indicated by a yellow star. Territories of surrounding males were estimated with the Voronoi approach and marked with a marssymbol. This female was tracked for 15 days. [file peerj-08-8920-s007.pdf]

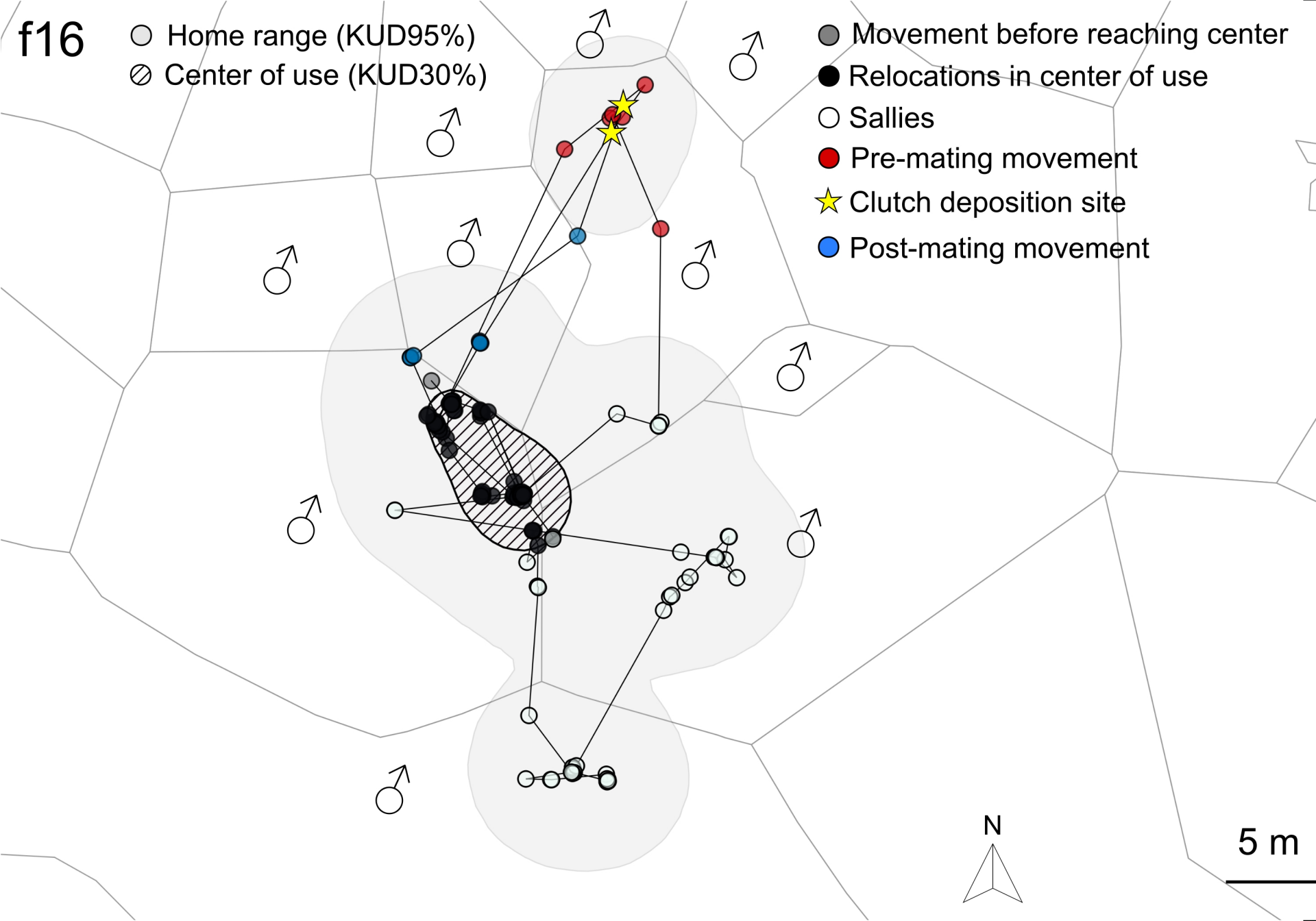

Supplement: Figure S12 — Female trajectory with one center of use. The center of use is striped (KUD30). HR area (KUD95) is shaded light grey. Relocalization points after tagging before reaching a center of use are shown in dark grey. Datapoints in the center of use are indicated in black, sallies to the surrounding are marked with hollow dots, pre-mating movement is indicated with red and post-mating movement until the next center of use is reached with blue dots. The egg deposition site is indicated by a yellow star. Territories of surrounding males were estimated with the Voronoi approach and marked with a marssymbol. This female was tracked for 17 days. [file peerj-08-8920-s016.pdf]

f19

- Home range (KUD95%)
- ◌ Center of use (KUD30%)

- Movement before reaching center
- Relocations in center of use

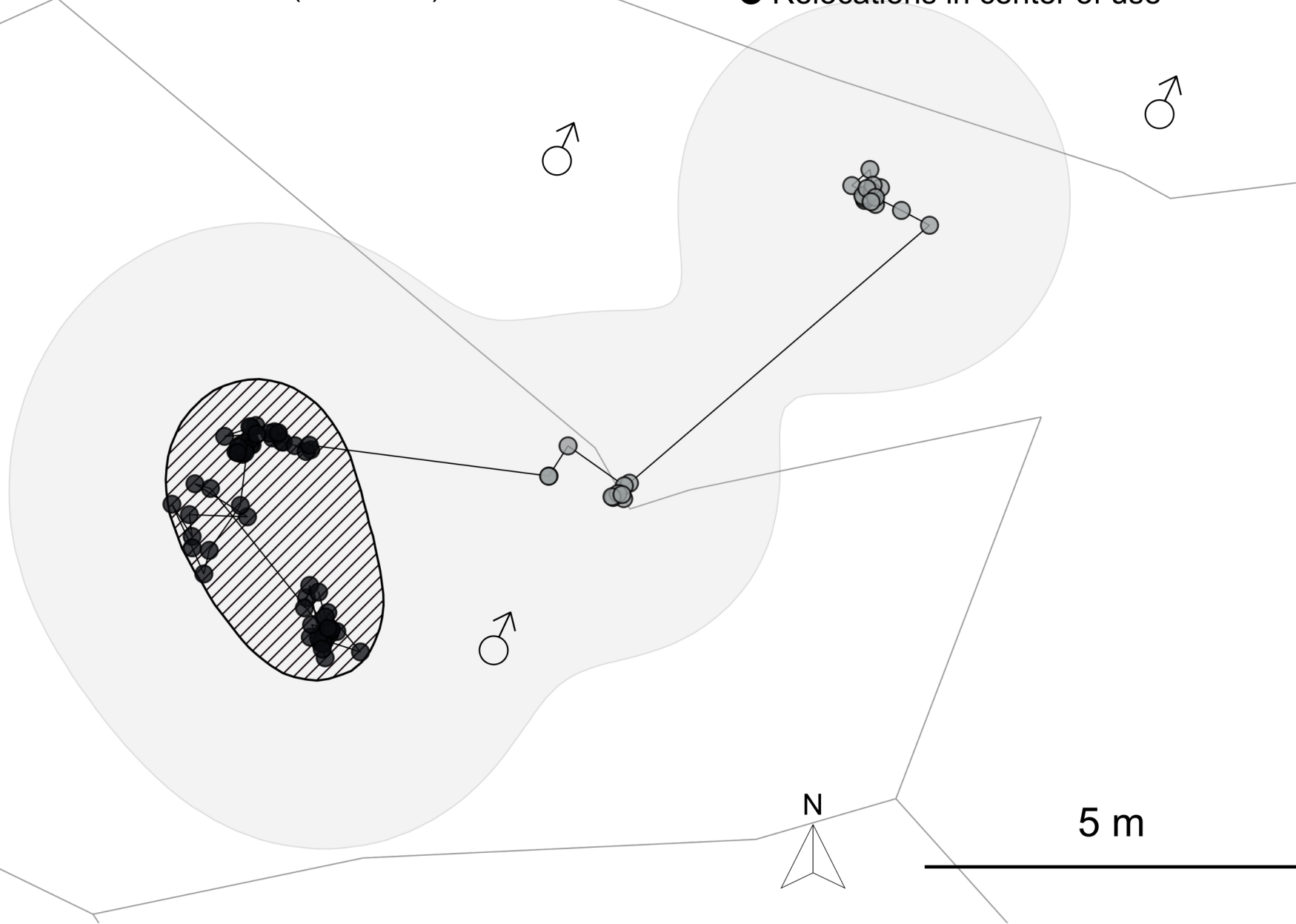

Supplement: Figure S14 — Female trajectory with one center of use. The center of use is striped (KUD30). HR area (KUD95) is shaded light grey. Relocalization points after tagging before reaching a center of use are shown in dark grey. Datapoints in the center of use are indicated in black. No sallies or courtship/mating events were observed for this female. Territories of surrounding males were estimated with the Voronoi approach and marked with a marssymbol. This female was tracked for ten days. [file peerj-08-8920-s018.pdf]

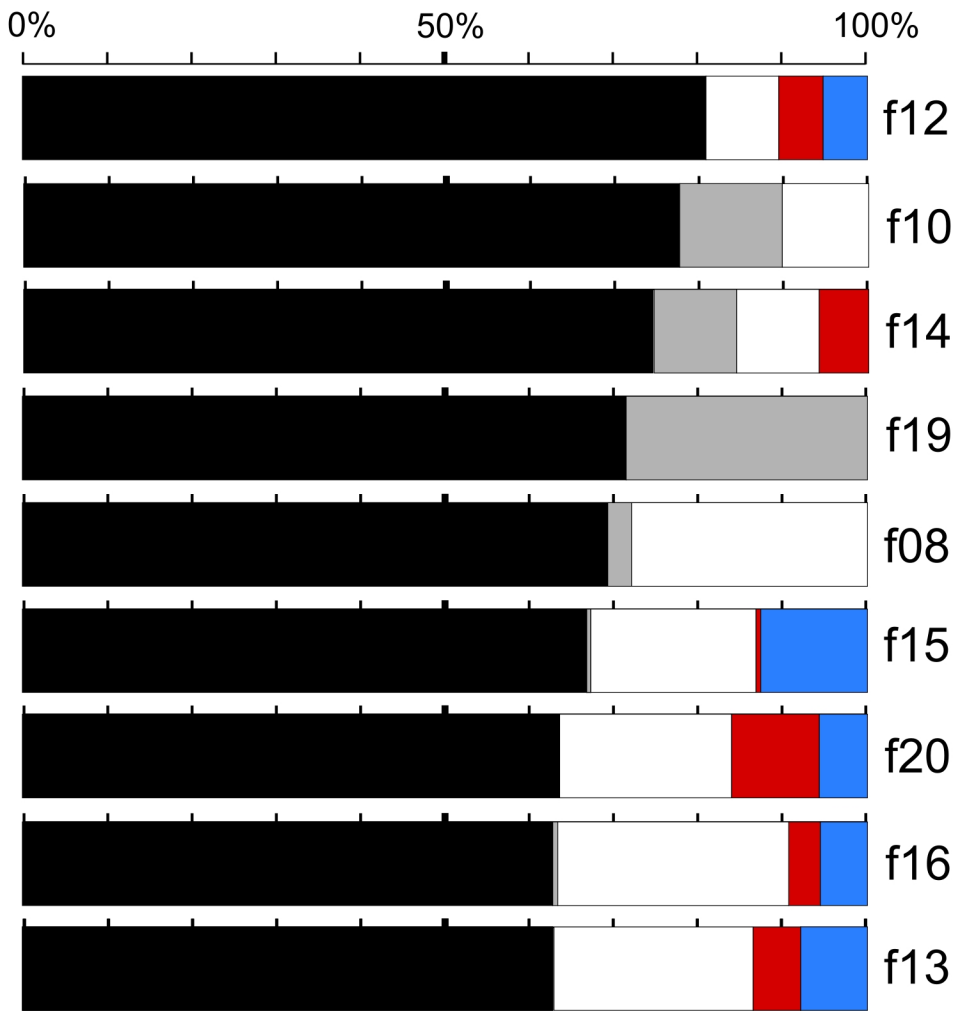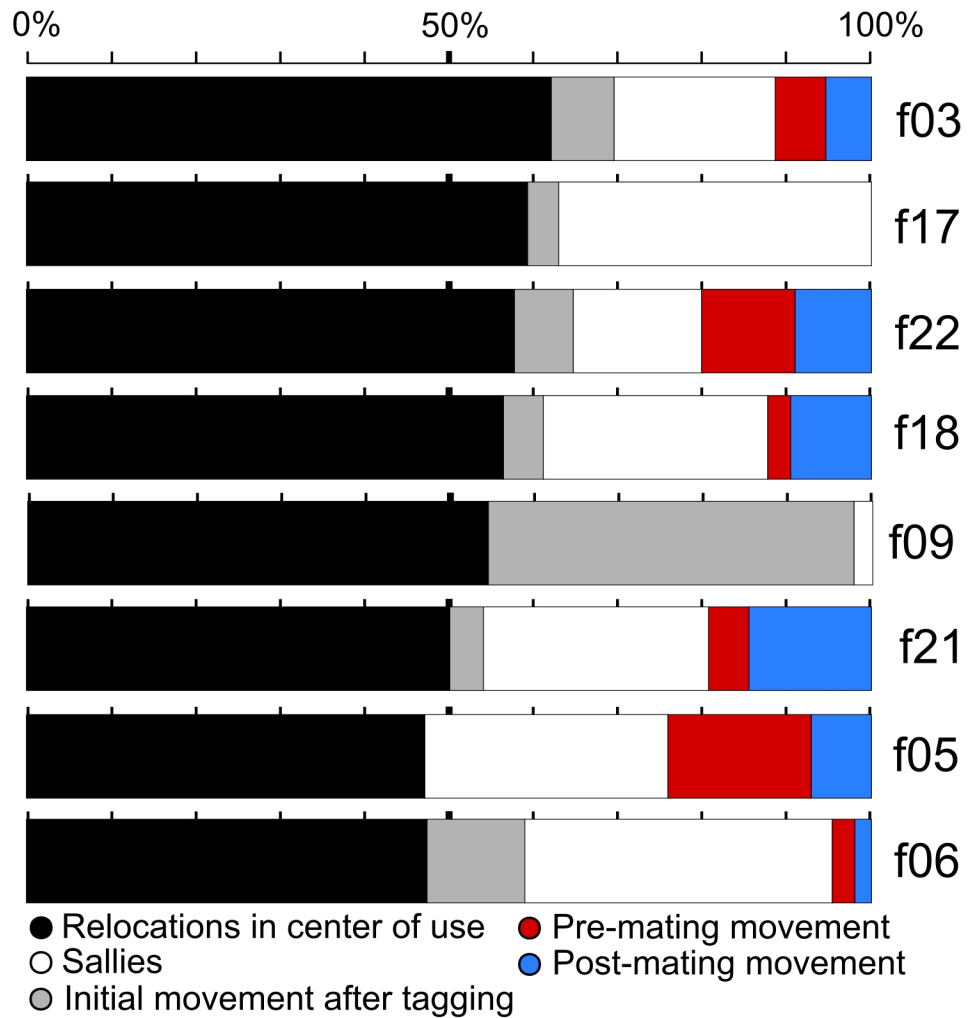

Supplement: Figure S18 — The percentage of time spent (i) on movement after tagging before reaching a center of use (grey), (ii) in the center of use (black), (iii) on sallies outside of the center (white), (iv) on pre-mating movement towards a mating partner (red) and (v) on post-mating movement until reaching the next center of use (blue) is illustrated. [file peerj-08-8920-s022.pdf]

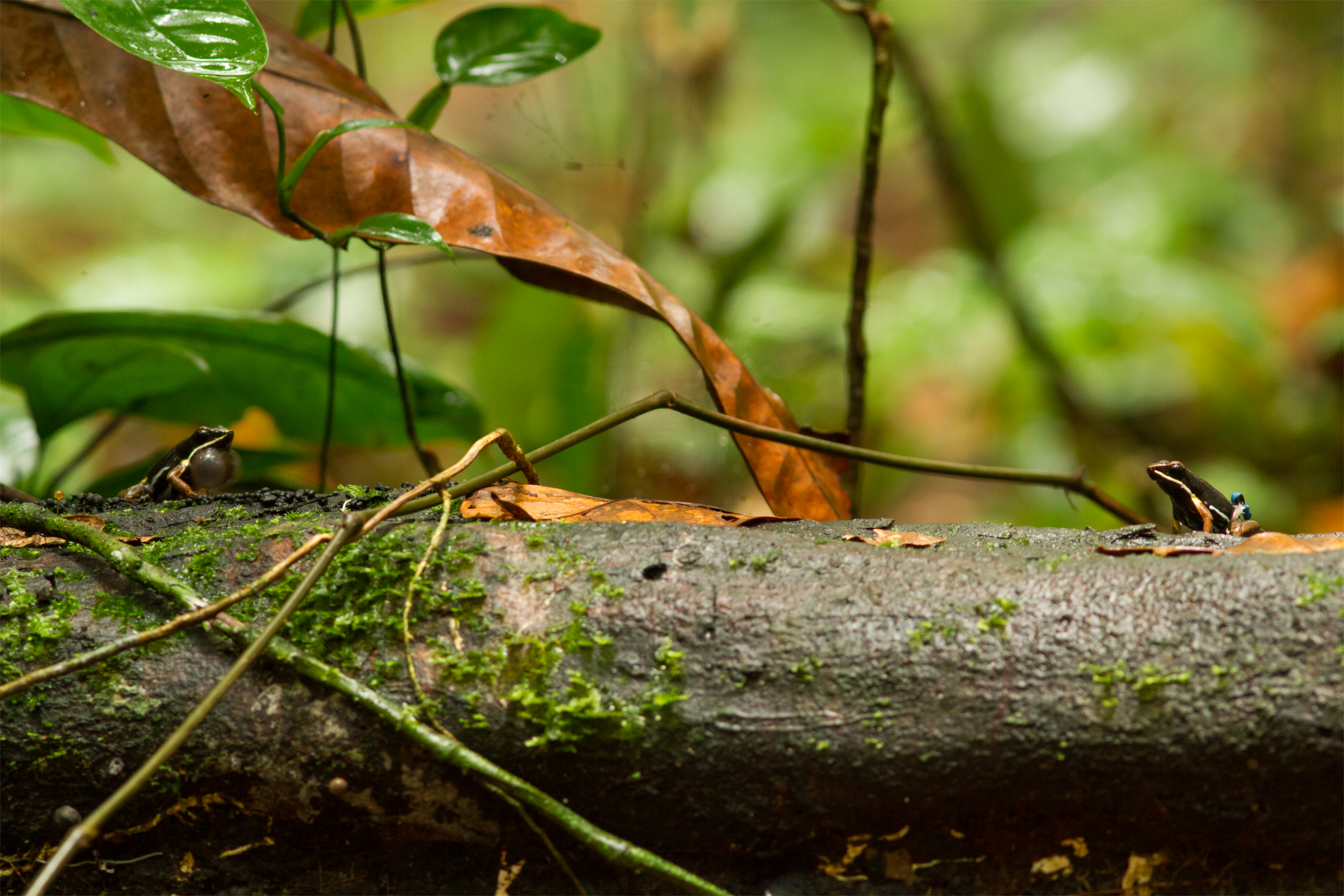

Supplement: Figure S19 — Female A. femoralis equipped with a tag (right side) attending a calling male (left side) after actively approaching him. Photo credit: Andrius Pašukonis. [file peerj-08-8920-s023.png]
