# Supplementary material for: Reproductive behavior drives female space use in a sedentary Neotropical frog
Source: PeerJ. 2020 Apr 17;8:e8920. doi: 10.7717/peerj.8920 (PMC7169969; doi:10.7717/peerj.8920)
Supplement: Figure S5 — Female trajectory with one center of use. The center of use is striped (KUD30). HR area (KUD95) is shaded light grey. Relocalization points after tagging before reaching a center of use are shown in dark grey. Datapoints in the center of use are indicated in black, sallies to the surrounding are marked with hollow dots, pre-mating movement is indicated with red and post-mating movement until the next center of use is reached with blue dots. The egg deposition site is indicated by a yellow star. This female was observed transporting one tadpole, as indicated with green datapoints. Territories of surrounding males were estimated with the Voronoi approach and marked with a marssymbol. This female was tracked for 16 days. [file peerj-08-8920-s009.pdf]

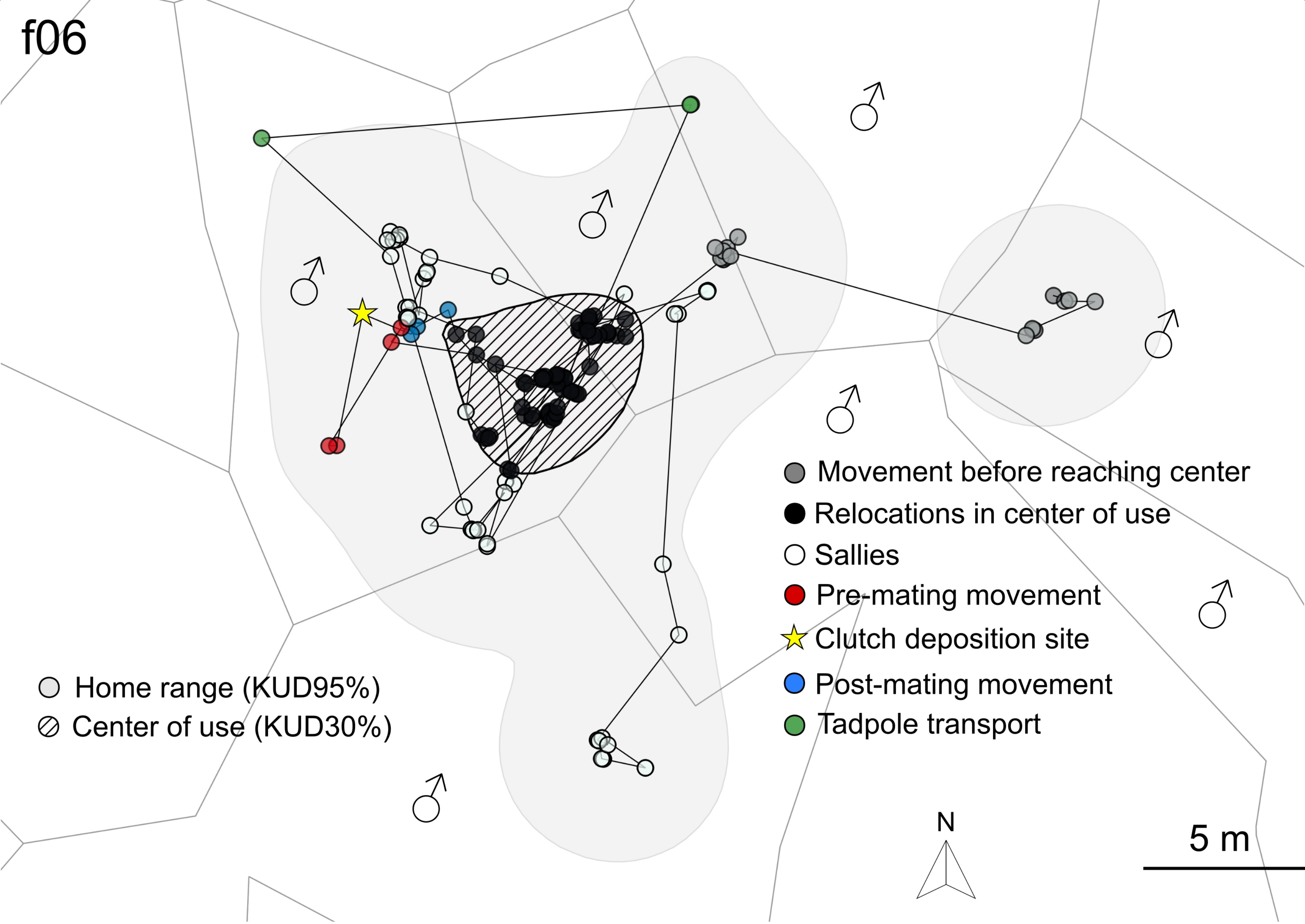
f06
Movement before reaching center
Relocations in center of use
Sallies
Pre-mating movement
Clutch deposition site
Post-mating movement
Tadpole transport
Home range (KUD95%)
Center of use (KUD30%)
N
5 m
