# Supplementary material for: Reproductive behavior drives female space use in a sedentary Neotropical frog
Source: PeerJ. 2020 Apr 17;8:e8920. doi: 10.7717/peerj.8920 (PMC7169969; doi:10.7717/peerj.8920)
Supplement: Figure S10 — Female trajectory with three centers of use. Centers of use (C1, C2, C3) are striped (KUD30). HR area (KUD95) is shaded light grey. Datapoints in the center of use are indicated in black, sallies to the surrounding are marked with hollow dots, pre-mating movement is indicated with red and post-mating movement until the next center of use is reached with blue dots. The egg deposition site is indicated by a yellow star. This female was tagged after oviposition. Territories of surrounding males were estimated with the Voronoi approach and marked with a marssymbol. This female was tracked for eight days. [file peerj-08-8920-s014.pdf]

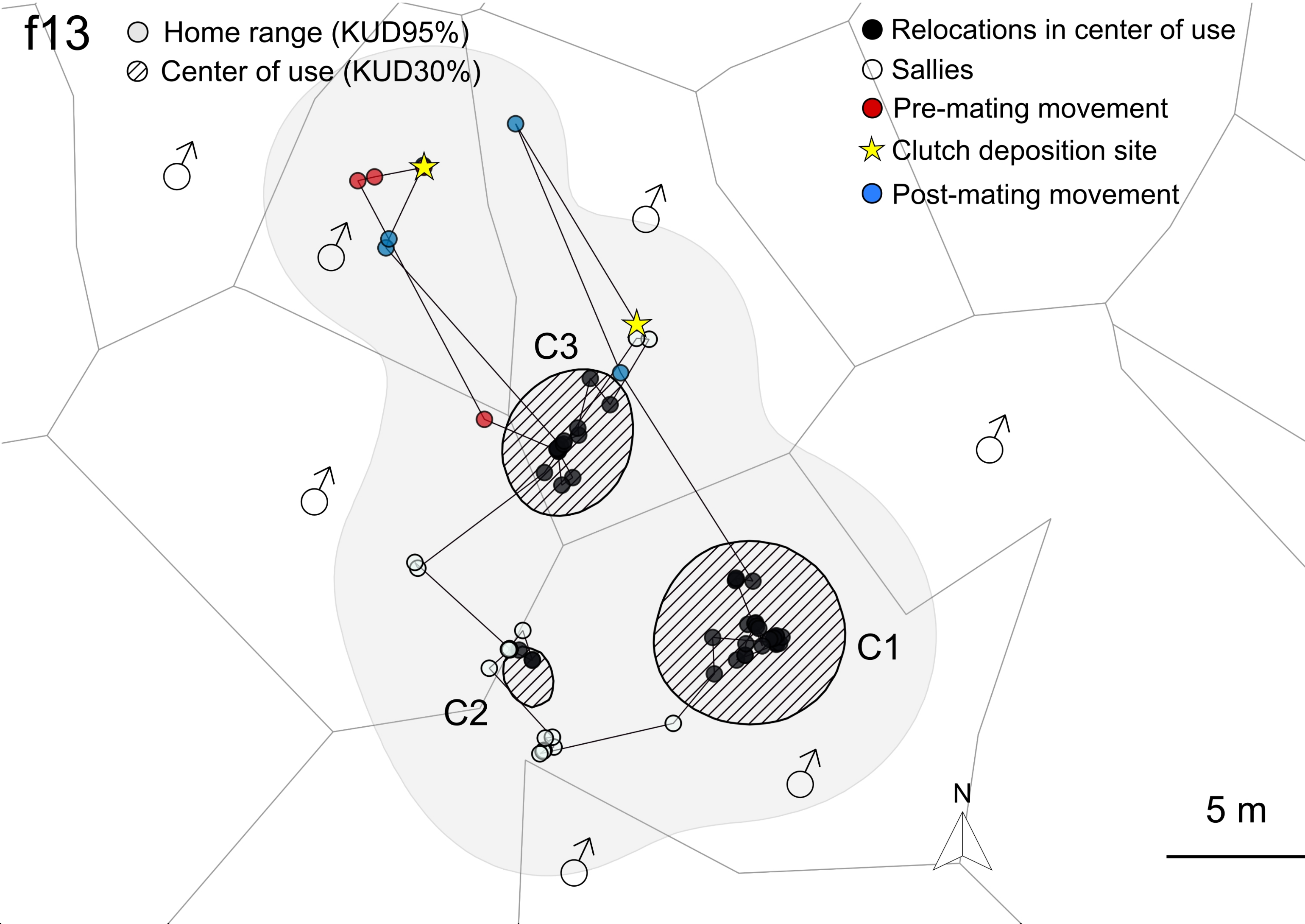

f13
Home range (KUD95%)
Center of use (KUD30%)
Relocations in center of use
Sallies
Pre-mating movement
Clutch deposition site
Post-mating movement
C3
C2
C1
N
5 m
