# Supplementary material for: Reproductive behavior drives female space use in a sedentary Neotropical frog
Source: PeerJ. 2020 Apr 17;8:e8920. doi: 10.7717/peerj.8920 (PMC7169969; doi:10.7717/peerj.8920)
Supplement: Figure S15 — Female trajectory with one center of use. The center of use is striped (KUD30). HR area (KUD95) is shaded light grey. Datapoints in the center of use are indicated in black, sallies to the surrounding are marked with hollow dots, pre-mating movement is indicated with red and post-mating movement until the next center of use is reached with blue dots. The egg deposition site is indicated by a yellow star. Territories of surrounding males were estimated with the Voronoi approach and marked with a marssymbol. This female was tracked for 17 days. [file peerj-08-8920-s019.pdf]

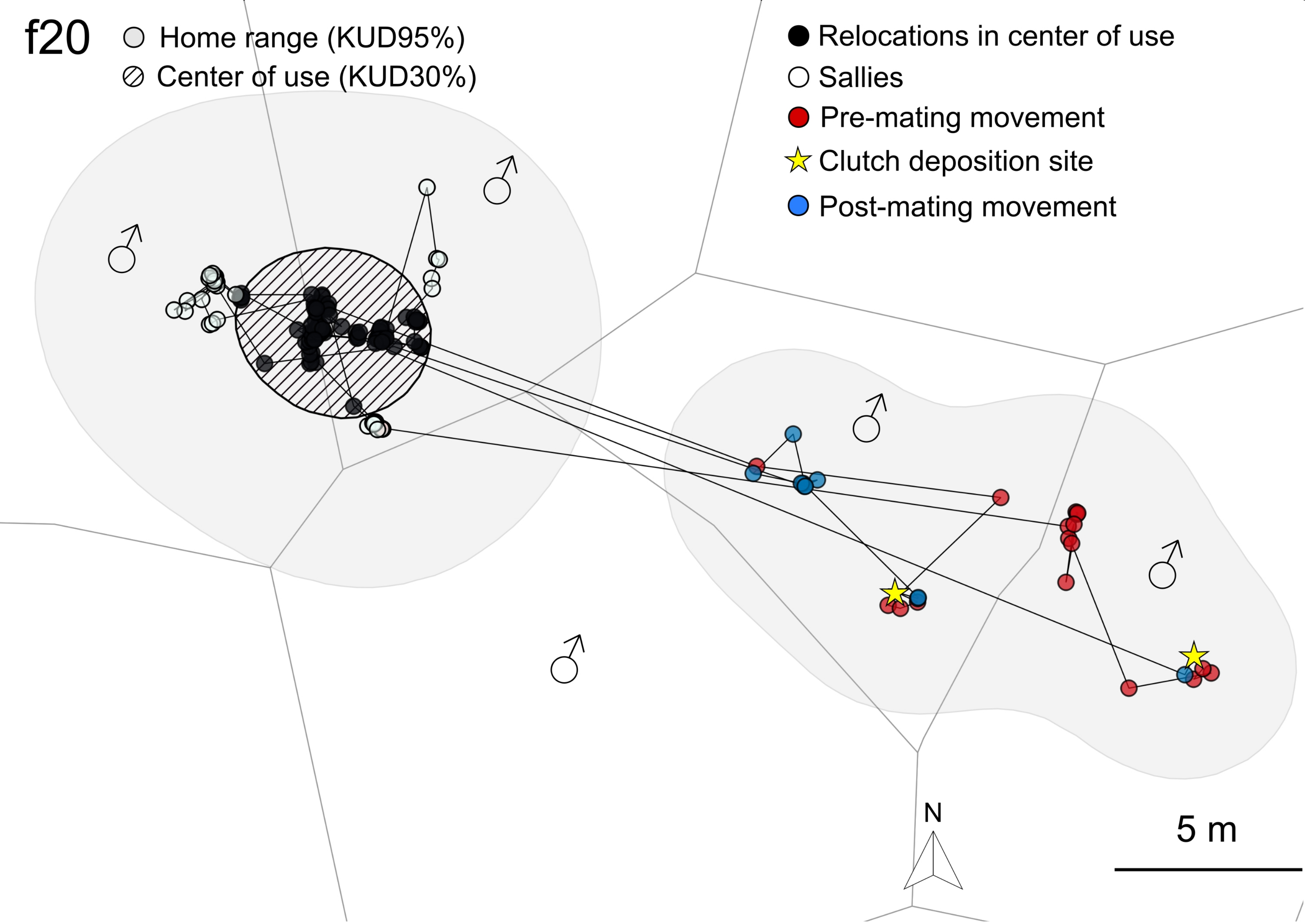

f20
Home range (KUD95%)
Center of use (KUD30%)
Relocations in center of use
Sallies
Pre-mating movement
Clutch deposition site
Post-mating movement
N
5 m
